# Identification of International Classification of Functioning, Disability and Health (ICF) codes most frequently used to describe functioning in children: a systematic review

**DOI:** 10.1136/bmjpo-2025-004292

**Published:** 2026-06-19

**Authors:** Idalina Maria Santos Vieira Lisboa Bordalo, Carla Martins Pereira, César Fonseca, Isabel Bico

**Affiliations:** 1 Health Technologies, Universidade NOVA de Lisboa Comprehensive Health Research Centre, Lisbon, Portugal; 2 Paediatrics, Unidade Local de Saúde Santa Maria, Lisbon, Portugal; 3 Directorate General of Health, Laranjeiro, Portugal; 4 Comprehensive Health Research Centre Évora Branch, Evora, Portugal

**Keywords:** Child Health, Child, Children, Health Policy

## Abstract

**Objectives:**

To identify and synthesise the International Classification of Functioning, Disability and Health (ICF) categories most used to describe child functioning across health conditions, informing inclusive, context-sensitive assessments and policy frameworks.

**Design:**

Systematic review.

**Data sources:**

PubMed, Scopus, Web of Science and CINAHL were searched from inception to September 2024 following Cochrane guidelines.

**Eligibility criteria:**

Peer-reviewed studies using ICF categories to assess functioning in children (0–18 years), regardless of health condition, were included. Eligible designs comprised randomised controlled trials, observational and qualitative studies reporting ICF Core Set (CS).

**Data extraction and synthesis:**

Two reviewers independently conducted screening, data extraction and risk of bias assessment using the Mixed Methods Appraisal Tool (MMAT, 2018). ICF codes were categorised by domain and synthesised narratively.

**Results:**

Eight studies met inclusion criteria, comprising 29 ICF CS (1665 instances; 194 unique ICF categories after deduplication). Most instances related to activities and participation (40.7%) and environmental factors (30.6%), followed by body functions (27.3%) and body structures (1.4%). Key chapters included learning and applying knowledge, interpersonal interactions, support and relationships and services and policies.

**Conclusions:**

Findings highlight a shift towards a biopsychosocial model of child functioning, emphasising participation and environmental context. Results support use of the ICF framework in multidisciplinary assessment and policy development and inform development of a Portuguese National Functioning Table for Children.

**PROSPERO registration number:**

CRD42024588533.

**WHAT IS ALREADY KNOWN ON THIS TOPIC**

- The International Classification of Functioning (ICF) is widely used in paediatric research, but synthesis of commonly applied categories across health conditions remains limited.

**WHAT THIS STUDY ADDS**

- This review identifies ICF categories most frequently used across paediatric Core Set, with predominance of activities and participation and environmental factors.

**HOW THIS STUDY MIGHT AFFECT RESEARCH, PRACTICE OR POLICY**

- Findings inform inclusive, diagnosis-independent assessment frameworks and support integrated approaches to paediatric care, education and policy.

## Introduction

 The ICF, developed by the WHO, provides a comprehensive framework, grounded in the biopsychosocial model of health, for describing human functioning across life domains and health conditions. This model conceptualises functioning and disability as dynamic outcomes arising from the interaction between biological, psychological and social factors.^1^ Rather than viewing disability as an inherent attribute of the individual, the ICF posits that it results from the interplay between a person’s health condition and contextual factors, such as environmental barriers and personal characteristics.^1^ The framework comprises three core components: body functions and structures, activities and participation, and contextual factors.^1^ Its holistic and integrative approach facilitates a nuanced understanding of health and disability, recognising that limitations in functioning often arise from mismatches between individual capabilities and environmental demands.^1^

Beyond its classificatory role, the ICF is increasingly used as a health outcome measure, supporting the evaluation of interventions by capturing real-life functioning. It is widely applied in clinical, public health, research and educational contexts, enabling standardised assessment, evidence-based policymaking, cross-cultural data comparability and inclusive, person-centred care.^2 3^

In Europe, the prevalence of chronic conditions among school-aged children varies substantially, ranging from approximately 8% to over 28% across countries, with higher rates observed in Northern Europe.^4^ Conditions such as paediatric inflammatory bowel disease have demonstrated rising incidence over the past five decades, with Crohn’s disease reaching rates of up to 10 per 100 000 children in certain regions.^5^ Neurodevelopmental conditions—including autism spectrum disorder, intellectual disability or sensory impairments—are also prevalent. The estimated prevalence of autism spectrum disorder in European children ranges from 0.38% to 1.55%, with evidence suggesting increasing trends.^6 7^ Mental health disorders represent a leading cause of disability-adjusted life years in this population, reflecting their significant impact on health and well-being.^8^ In line with the ICF framework, disability does not necessarily imply disease, but reflects the interaction between functioning and contextual factors. Chronic conditions and disabilities not only affect physical health but also negatively influence educational experiences and life satisfaction. Children with chronic conditions report lower school satisfaction, more negative school experiences and diminished global well-being compared with their peers.^4^ Overall, while the prevalence and types of chronic conditions and disabilities vary across Europe, their impact on children’s daily lives, education and mental health is significant and growing.^4^,^8^ These findings underscore the urgent need for inclusive policies and integrated support systems.

Children with multiple comorbidities demonstrate greater healthcare utilisation, including more frequent outpatient visits, hospitalisations and reliance on medical technologies, placing disproportionate demand on healthcare systems.^9 10^ In Spain, for instance, children with complex chronic conditions averaged over 15 healthcare interactions annually, with significantly higher utilisation among those with multiple diagnoses or palliative care needs.^10^ While most children with multiple conditions exhibit only modest increases in healthcare use, a small subset—often older, female and from lower income households—are high users, reflecting the intersection of clinical complexity and social factors.^9^ These patterns highlight persistent gaps in care coordination and reinforce the need for equitable, biopsychosocial models of care that address both clinical and socioeconomic dimensions.^9 10^

To enhance the practical applicability of the ICF framework, condition-specific ICF Core Set (CS) were developed through a rigorous, multiphase process involving systematic literature reviews, empirical studies, expert surveys and consensus conferences.^11^ These CS provide concise, condition-relevant selections of ICF categories, allowing for structured and comprehensive assessment of functioning in targeted populations. However, their scope is often limited to single diagnostic categories, reducing their utility in inclusive settings where children present with multiple or co-occurring conditions. This diagnosis focus limits their applicability in population-based and preventive contexts, where child functioning must be assessed independently of diagnosis. In many health and education systems, there is a pressing need for a unified, transdiagnostic framework to assess child functioning in a manner that is developmentally appropriate, equitable. Despite this need, no nationally validated, diagnosis-independent reference supports systematic classification of functioning across paediatric populations.

Although condition-specific ICF CS enables structured assessment in defined clinical populations, many paediatric settings require a complementary cross-condition (‘generic’) set. In childhood, diagnosis is often evolving, multiple or uncertain, with frequent comorbidity. In early intervention, inclusive education, rehabilitation, primary care and community services, support and goal setting are often guided by functional needs rather than diagnostic labels. A cross-condition set would provide a shared language for multidisciplinary teams, facilitate comparability across services and studies and inform policy and resource planning based on functioning.

The relevance and interpretation of ICF domains differ in childhood, as functioning is embedded in developmental trajectories and age roles. Across 0–18 years, rapid change occurs in learning, play, communication, autonomy, schooling and peer relationships, and environmental facilitators and barriers (family, school, services and policies) critically shape participation. Thus, while ICF categories are not child-exclusive, their prioritisation in paediatrics is developmentally anchored; identifying categories recurring across paediatric ICF CS offers an empirical basis for a child-focused cross-condition set.

To date, no systematic review has identified the ICF categories most relevant to the classification of children’s functioning—across body functions and structures, activities, participation and contextual factors—regardless of diagnosis. Existing reviews and CS focus primarily on specific conditions, such as autism spectrum disorder, attention-deficit/hyperactivity disorder, cerebral palsy or rare syndromes.^212^,^15^ While these studies highlight the importance of domains such as activities and participation, body functions and—less frequently—environmental factors, their scope remains condition-specific. A recent systematic review of paediatric ICF-CY CS confirmed that only 12 sets currently exist, mostly targeting specific health conditions, emphasising the need for broader, more inclusive tools applicable across paediatric populations.^12^ Although a CS for child development has been developed,^16^ evidence show that no single instrument or existing CS comprehensively captures all domains of child functioning, indicating a persistent gap in population-level assessment.^17^

This systematic review is part of a broader initiative aimed at developing a national, diagnosis-independent functioning reference for children, focusing on ages 0–18 to capture developmental trajectories and age-specific functional competencies rather than static abilities. By identifying the ICF categories most frequently used to describe children’s functioning, this review provides an empirical foundation for a harmonised, transdiagnostic classification tool, intended to support population monitoring, identification of unmet health needs, and longitudinal follow-up using functioning as a core health indicator.

### Objective

This systematic review aims to identify the ICF categories across body functions and structures, activities, participation and contextual factors that are most relevant to the classification of children’s functioning, independent of health condition. The ultimate goal is to generate a comprehensive list of ICF codes that reflect the domains used to characterise children’s functioning in a universal and inclusive manner. These findings will serve as a foundational step for the subsequent consensus and validation phases of development of a national functioning table for children, intended to support standardised assessments, enhance intersectoral coordination and inform inclusive policymaking.

This review has been conducted in accordance with the Preferred Reporting Items for Systematic Reviews and Meta-Analyses (PRISMA) guidelines^18^ to ensure methodological transparency and rigour.

## Methods

This systematic review was conducted and reported in accordance with the PRISMA 2020 guidelines^18^ to ensure transparency, completeness and methodological rigour. A completed PRISMA checklist^19^ is provided as an online supplemental file. The review protocol was prospectively registered with PROSPERO (registration number: CRD42024588533) and accepted in September 2024. The protocol is publicly available on the PROSPERO website.

### Literature search

A comprehensive literature search was performed across four electronic databases: PubMed, Scopus, Web of Science and CINAHL. Following Cochrane recommendations,^20^ all records from inception to September 2024 were included. The primary search string included: (“International Classification of Functioning, Disability and Health”[Mesh] OR “Disability Evaluation”[Mesh] OR “ICF”) AND (“core set” OR “CS” OR “code set” OR “code sets”), (“International Classification of Functioning, Disability and Health”[Mesh] OR “Disability Evaluation”[Mesh] OR “ICF”) AND (“core set” OR “CS” OR “code set” OR “code sets”) AND (“Child”[Mesh] OR “Adolescent”[Mesh] OR “Child, Preschool” OR “Infant” OR “Infant, Newborn”[Mesh]). Search strategies were adapted for each database using appropriate indexing terms and filters. Full database-specific strategies are provided in the online supplemental materials.

### Eligibility criteria and data extraction

The aim of this review was to identify the ICF categories—across body functions and structures, activities and participation and contextual factors—that are most relevant to the classification of children’s functioning, irrespective of health condition. Eligibility criteria were defined using a Population, Intervention, Comparison, Outcome framework: two independent reviewers screened titles and abstracts, extracted data using a structured form, and categorised ICF codes by component.

#### ICF category handling and definitions

For each included publication, all ICF category listed in each reported CS (brief or comprehensive; lifespan or age-specific) were extracted. To avoid ambiguity in reporting, we distinguished between: (1) code instances—the total number of ICF categories contained in the identified CS summed across CS (reflecting the content volume) and (2) unique ICF categories—distinct ICF categories after removing duplicates. Categories were grouped by ICF component and chapter. Frequency was defined as the number of CSs in which each unique category appeared; the complete list and frequencies appeared; the complete list of frequencies is provided in online supplemental material.

#### Population

Children and adolescents aged 0–18 years with any health condition.

#### Intervention

Assessment or use of ICF.

#### Comparison

Not applicable.

#### Outcome

List of ICF codes reported and/or used to characterise children’s functioning.

#### Study design

Randomised controlled trials, controlled clinical trials, observational studies (cross-sectional and longitudinal) and qualitative studies published in peer-reviewed journals. Eligible studies were written in English, Portuguese, French or Spanish.

Exclusion criteria included study protocols, case reports, conference abstracts, economic evaluations, psychometric or prevention studies, phase II clinical trials, laboratory-based studies, grey literature, letters, editorials and commentaries.

Two independent reviewers (IMSVLB and CMP) screened titles and abstracts and selected articles for full-text review. During full-text screening, the reviewers independently extracted all relevant ICF codes mentioned or applied in the studies.

Data were extracted using a custom-designed, Excel-based data form piloted on the first five included studies. To calibrate inter-reviewer consistency, the first 10 references were independently reviewed and discussed. The form was iteratively refined to capture relevant variables not anticipated a priori. Extracted data included: bibliographic details (authors, year, title, DOI and journal); study design; participant characteristics (age group, health condition); methodology used to develop ICF CS; type of CS (comprehensive or brief); list of ICF codes used or referenced, categorised by component. Data extraction was performed independently by the two reviewers, who were blinded to each other’s decisions.

### Risk of bias assessment

Methodological quality and risk of bias of the included studies were assessed using the Mixed Methods Appraisal Tool (MMAT V.2018)^21^ following its standard guidance. The MMAT is designed for qualitative, quantitative and mixed method studies in systematic reviews and has demonstrated validity and reliability.^18 19^ Two reviewers (IMSVLB and CMP) conducted the assessments independently, and discrepancies were resolved by discussion or consultation with a third reviewer (IB, CF). The results of the MMAT assessments are summarised in tabular form and considered during data synthesis and interpretation. The full MMAT checklist is provided as online supplemental material.

### Data synthesis and presentation

The primary objective of the synthesis was to identify and summarise the ICF codes most commonly used to characterise children’s functioning across published paediatric CS. Due to the descriptive nature of the data and heterogeneity of study designs, health conditions and measurement approaches, no meta-analysis was conducted. Consequently, standard effect measures (eg, risk ratios, mean differences) were not calculated.

Studies were grouped based on the ICF component(s) addressed: body functions, body structures, activities and participation and environmental factors. All ICF codes included in eligible CS were retained without a priori selection and compiled into structured tables, categorised according to the ICF hierarchy. Frequencies of occurrence across CS were calculated to identify the most prevalent categories within each component. No imputation or data transformation procedures were applied.

Given the wide variability in populations and study aims, no subgroup analyses, assessments of statistical heterogeneity or sensitivity analyses were undertaken. Formal assessment of evidence certainty (eg, using GRADE) was not conducted, as the review focused on descriptive mapping rather than the measurement of intervention effects.

## Results

### Study selection

The database search was completed in September 2024. A total of 396 records were identified across four databases: MEDLINE, CINAHL, Scopus and Web of Science. After removing 180 duplicates and 4 ineligible, 212 records remained for screening. Titles and abstracts were assessed for eligibility, resulting in the exclusion of 75 records. The full texts of the remaining 126 articles were reviewed in detail. Of these, 81 were excluded as preparatory studies, 18 were excluded due to lack of available ICF CS codes and 26 for being case reports.

**Figure 1:**
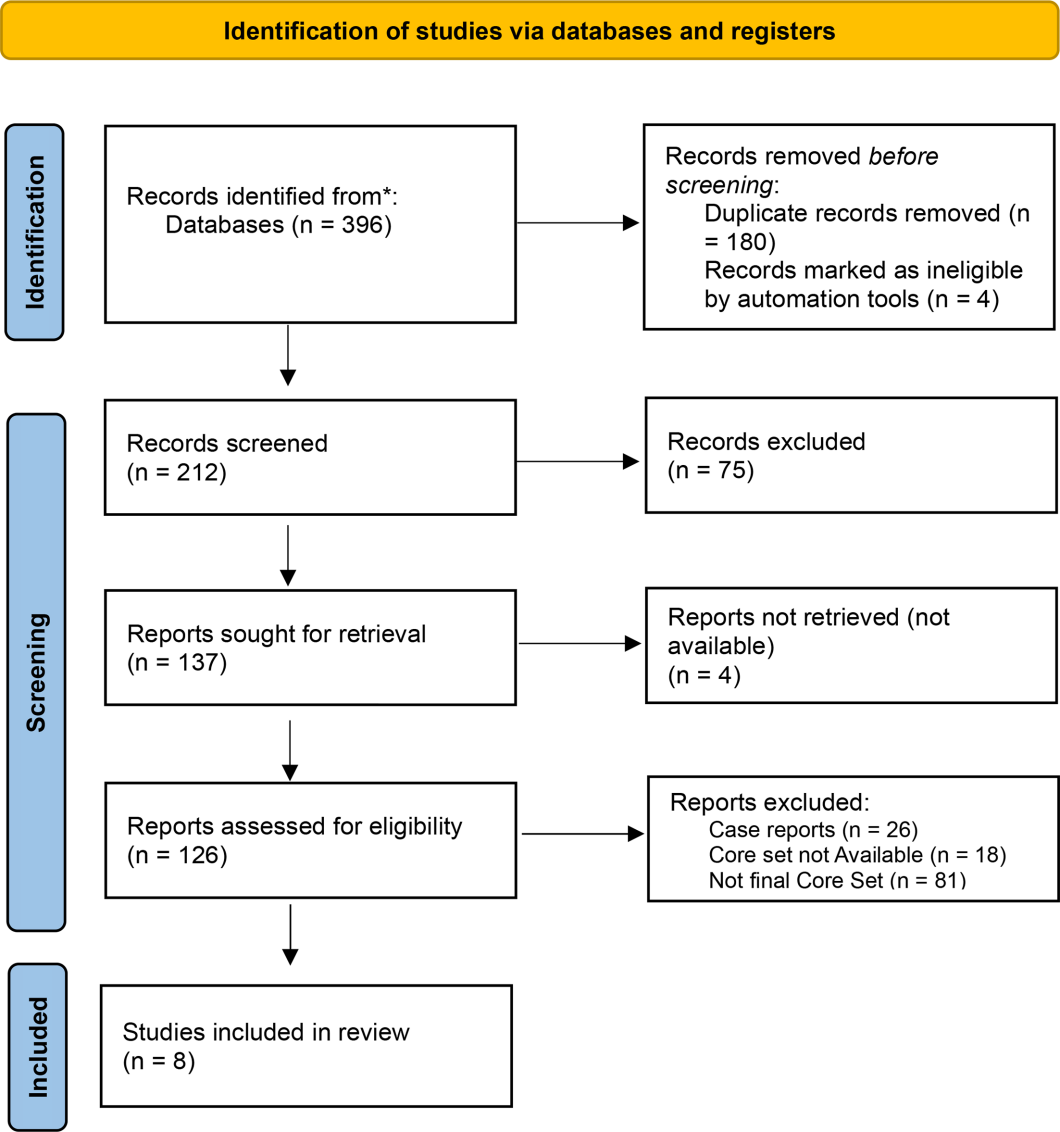
PRISMA Flow diagram^18^ for search and screening process.

A final total of eight studies met the inclusion criteria and were included in the qualitative synthesis. The full study selection process is illustrated in the PRISMA flow diagram (figure 1), in accordance with PRISMA 2020 guidelines.^18^

### Study characteristics

For each included study, the following data were extracted: bibliographic details; study design; participant characteristics (age group and health condition); methodology used to develop or apply ICF CS; type of CS (comprehensive or brief) and the list of ICF codes reported, categorised by component (body functions, body structures, activities and participation and environmental factors). An overview of the characteristics of the included studies is presented in table 1.

**Table 1:** Summary of characteristics of included studies (N=8)

Study (author, year)	Country/setting	Study design	Methodology	Focus/clinical area	Participants	ICF CS/age group
Luo *et al*, 2022^28^	China; hospital setting (postsurgery)	Mixed-methods study	Systematic review, qualitative interviews (experts and parents), two-round Delphi expert consensus, Rasch analysis, validation study	Cardiac rehabilitation post-CHD surgery	270 children (3–6 years post-CHD surgery); 36 experts; 18 parents; 14 medical staff	ICF-CY CS for cardiac rehabilitation 3–6 years)
Güeita-Rodríguez *et al*, 2019^26^	International (21 countries); aquatic therapy clinics, community settings	Delphi consensus study	Review of prior preparatory phase (systematic review, Delphi three-round survey, qualitative focus groups/interviews with parents), final Delphi consensus	Neurological conditions, rehabilitation (aquatic physical therapy)	69 experts; parents (n=43)	Aquatic physical therapy core set; comprehensive 0–18; common brief; 0 -<6 years; >6–<14 year; >14–<18 years
Bölte *et al*, 2024 (Autism revision)^22^	Sweden, UK, Germany, Australia, Austria; clinical and community settings	Delphi-like iterative process	Validation/linking studies, stakeholder feedback, pilot testing, translation	Autism spectrum disorder (ASD)	13 experts; autistic individuals, relatives, professionals	Autism ICF CS (revision); comprehensive/lifespan; comprehensive 0–5 years comprehensive 6–16 years
Bölte *et al*, 2019 (Autism original)^24^	International; clinical settings	Consensus conference	review of prior four preparatory studies (systematic review, expert survey, qualitative study, clinical study)	ASD	29 international experts	Autism ICF CS (original); comprehensive/ lifespan; brief/ lifespan; brief/0–5 years; brief 6A 16 years
Bölte *et al*, 2024 (ADHD revision)^23^	Sweden, Germany, Austria, Australia; clinical and community settings	Delphi-like iterative process	Stakeholder feedback, pilot testing, review of prior preparatory studies—expert survey, scoping review, clinical study, qualitative study	Attention deficit hyperactivity disorder (ADHD)	ten experts; ADHD individuals and caregivers	ADHD ICF CS (revision); comprehensive/ lifespan; brief/lifespan; brief/0–5 years; brief 6–16 years
Bölte *et al*., 2018 (ADHD original)^25^	International; clinical settings	Consensus conference	Review of prior four preparatory studies—scoping review, expert survey, qualitative study, clinical study	ADHD	30 international experts	ADHD ICF CS (original); comprehensive/lifespan; 0–5 years; 6–16 years
Schiariti *et al*, 2015^27^	Canada, international experts; rehabilitation clinics, community settings	WHO-endorsed methodology+consensus conference	review of prior four preparatory studies (systematic review, international survey, qualitative interviews, clinical study); operationalising ICF-CY CS; linking ICF-CY codes to functional indicators	Cerebral palsy (CP), paediatric rehabilitation	29 experts; two parents	ICF CS for children and youth with cerebral palsy; brief 0–18 years; brief 0–6 years; brief 6–14 years; brief 14–18 years comprehensive 0–18 years
Ellingsen & Simeonsson, 2024^16^	International (27 countries)	Delphi	Four rounds Delphi Literature review	Child development	151 experts	Developmental core set 0–2 years 3–5 years 6–12 years 13–17 years

CS, Core Set; ICF, International Classification of Functioning, Disability and Health.

Most studies^22 23^ applied the ICF Research Branch Methodology,^11^ which comprises three phases: (1) collection of evidence through four preparatory studies (systematic review, expert survey, clinical study, and qualitative research); (2) expert consensus conference to finalise codes; and (3) implementation of the resulting CS. Three studies employed consensus methods, such as Delphi procedures or expert conferences, without the full ICF Research Branch framework. Two of these were updates of prior versions,^22 23^ and one developed a CS via a large international Delphi panel.^16^

### CS and ICF code distribution

Across the eight included studies, a total of 29 ICF CS were identified, including:

Seven life-span CS (comprehensive or brief).^22^,^25^

Two sets for children and adolescents aged 0–18 years.^26 27^

Nineteen age-specific CS across various health conditions.^1622^,^28^

These CS comprised a total of 1665 ICF code instances across CS. After deduplication, 194 unique ICF categories were identified. When considering code instances (ie, the content volume of CS), categories were distributed as follows: activities and participation (40.72%), environmental factors (30.57%), body functions (27.27%) and body structures (1.4%). This distribution highlights the emphasis on daily functioning and contextual aspects in existing ICF classifications for children. A summary of the frequency and distribution of ICF codes by component and CS is presented in table 2, and the detailed codes list as online supplemental information.

**Table 2:** Frequency and distribution of ICF codes across included CS (N=29)

ICF component	Code instances (sum across CS)	Percentage
Activities and participation	678	40.72
Learning and applying knowledge	166	
General tasks and demands	74	
Communication	65	
Mobility	83	
Self-care	78	
Domestic life	22	
Interpersonal interactions and relationships	87	
Major life areas	71	
Community, social and civic life	32	
Environmental factors	509	30.57
Products and technology	98	
Natural environment and human-made changes to environment	22	
Support and relationships	136	
Attitudes	139	
Services, systems and policies	114	
Body functions	454	27.27
Mental functions	225	
Sensory functions and pain	82	
Voice and speech functions	12	
Functions of the cardiovascular, haematological, immunological and respiratory systems	36	
Functions of the digestive, metabolic and endocrine systems	18	
Genitourinary and reproductive functions	3	
Neuromusculoskeletal and movement-related functions	77	
	1	
Body structures	24	1.4
Structures of the nervous system	6	
Structures involved in voice and speech	1	
Structures related to movement	17	
Total	1665	100

Counts represent the total categories per CS (code instances). Duplicates were removed only when calculating unique ICF categories (n=194).

CS, Core Set; ICF, International Classification of Functioning, Disability and Health.

## Discussion

### Summary of key findings

This systematic review identified the ICF categories most frequently used to describe the functioning of children, independent of health condition. From eight included studies, 29 ICF CS were retrieved, comprising 1665 1665 code instances and 194 unique ICF categories after removing duplicates. The majority of codes belonged to the domains of activities and participation (40.7%) and environmental factors (30.6%), with fewer codes related to body functions (27.3%) and body structures (1.4%). Within activities and participation, the most represented chapters were *Learning and applying knowledge*, *Interpersonal interactions and relationships* and *Self-care*. In environmental factors, the most prevalent chapters were *Support and relationships*, *Attitudes*, and *Services, systems and policies*.

These results reflect the adoption of a biopsychosocial model of child functioning, recognising the influence of environmental and contextual elements alongside individual health characteristics.

### Comparison with existing evidence

Our findings align with previous research advocating for a broader, holistic conceptualisation of childhood disability and development.^29^,^31^ The inclusion of codes related to participation and environmental is consistent with the WHO’s emphasis on person-centred inclusive care.^1^ The ICF framework, particularly in its child-focused version (ICF-CY), promotes a comprehensive understanding of functioning that incorporates both individual and contextual dimensions.^16^

This perspective is reflected in the ‘F-words framework’—*function, family, fitness, fun, friends* and *future*—which operationalises ICF principles into practical, family-centred approaches.^32^,^34^ The use of ICF CS in the included studies illustrates how the framework can guide both clinical decision-making and service planning, despite challenges in operationalisation.^12 29 30^

### Implications for practice and policy

The prominence of activities and participation codes highlights ICF’s relevance for multidisciplinary assessment and goal-setting in health and educational contexts. These domains cover critical daily life areas such as communication, mobility and social interaction, which are closely linked to quality of life. The substantial representation of environmental factors underscores the role of family, school and community contexts in enabling or restricting participation.

In practice, a child-focused cross-condition set derived from recurring categories across paediatric CS may be particularly valuable where diagnosis is uncertain, evolving or multiple, and where service eligibility and planning are functioning-based (eg, early intervention, inclusive education, rehabilitation and community programmes). It would complement, not replace, condition-specific CS; by enabling consistent documentation, multidisciplinary communication, comparable outcome monitoring and needs-based policy planning.

Sociocultural contexts shape how the ICF is interpreted and applied. For example, studies have shown that cultural values influence which ICF categories are considered relevant or meaningful.^35^ Implementation challenges arise when key environmental or social aspects are under-represented.^35 36^ Nonetheless, the ICF supports a shift from impairment-focused models towards inclusive approaches that reflect children’s lived experiences.

At the policy level, the ICF provides a standardised language facilitating integrated care across health, education, and social sectors.^16 37 38^ In countries where its use is formally mandated, such as Portugal, ICF-based documentation has contributed to more individualised assessments and support planning.^39^ Focusing on children aged 0–18 allows policies and services to reflect developmental trajectories and age-specific functional competencies, ensuring stage-appropriate maximal functional gains. Age and condition-specific CS further supports policy implementation, although continued efforts are needed to ensure tools are reliable, culturally appropriate and feasible in practice.^12 38^

The ICF framework has potential for standardising disability data, improve service coordination and support compliance with international disability rights conventions. The CS identified in this review can guide development of inclusive services and resource allocation based on functional needs rather than diagnosis alone.

### Future research directions

Several gaps were identified. Few CS offered a universal or transdiagnostic scope, and only a limited number of studies incorporated direct input from children and families. Future research should prioritise participatory methodologies and develop CS that are inclusive, culturally adaptable, and applicable across health conditions. There is also a need for continued refinement of ICF-based tools that are feasible for use in varied clinical and policy contexts.

## Conclusion

This review provides a comprehensive synthesis of ICF codes used to characterise child functioning across multiple CS. The dominance of participation-related and context-related codes underscores the importance of a holistic, interdisciplinary approach to paediatric assessment. These findings have implications for clinical practice, policy development and future research, supporting the continued evolution of child-focused ICF tools that are inclusive, functional and applicable. In Portugal, these results provide a foundational evidence for developing a national child functioning table, contributing to the standardisation and improvement of disability assessment and service planning for children.

## Supplementary material

10.1136/bmjpo-2025-004292 online supplemental file 1

10.1136/bmjpo-2025-004292 online supplemental file 2

10.1136/bmjpo-2025-004292 online supplemental file 3

10.1136/bmjpo-2025-004292 Abstract translation 1 This web only file has been produced by the BMJ Publishing Group from an electronic file supplied by the author(s) and has not been edited for content.

## Data Availability

All data relevant to the study are included in the article or uploaded as supplementary information.
