# Supplementary material for: Identification of International Classification of Functioning, Disability and Health (ICF) codes most frequently used to describe functioning in children: a systematic review
Source: BMJ Paediatr Open. 2026 Jun 19;10(1):e004292. doi: 10.1136/bmjpo-2025-004292 (PMC13289332; doi:10.1136/bmjpo-2025-004292)
Supplement: online supplemental file 1 [file bmjpo-10-1-s001.docx]

The PRISMA 2020 reporting checklist

For checking that systematic review articles can be understood and used by everyone

| 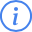 Note |
| --- |
| If you have not used a reporting guideline before, read about [how and why to use them](https:/resources.equator-network.org/about/reporting-guidelines.html) and check whether PRISMA 2020 is the [most applicable reporting guideline](https:/resources.equator-network.org/reporting-guidelines/prisma/index.html?#applicability) for your work.  Reporting guidelines are most useful when used early in research. When writing a manuscript or application, consider using the [Full Guidance](https:/resources.equator-network.org/reporting-guidelines/prisma/index.html) where you’ll see explanations and examples for each item.  After writing, demonstrate adherence by completing this checklist:   1. Specify where each item is described (see [Note 1](#sec-specify)). 2. Cite this checklist (See [Note 2](#sec-cite)). 3. Include your completed checklist as a supplement when submitting to a journal so that future readers can use it to find information. |

|  | Item Description | Location (or reason for not reporting) |
| --- | --- | --- |
| **Title and Abstract** |  |  |
| [1. Title](https:/resources.equator-network.org/reporting-guidelines/prisma/items/title.html?utm_source=prisma&utm_medium=checklist&utm_campaign=1_1) | Identify the report as a systematic review. | Title Page - Title: *“Child Functionality Through the Lens of the ICF: A Systematic Review of Paediatric Core Sets”* |
| [2. Abstract](https:/resources.equator-network.org/reporting-guidelines/prisma/items/abstract.html?utm_source=prisma&utm_medium=checklist&utm_campaign=1_1) | Include all items from the *PRISMA 2020 for Abstracts* checklist. | Abstract section - paragraphs 1 to 6 *(Objectives, Design, Data sources, Eligibility criteria, Data extraction and synthesis, Results, Conclusions, PROSPERO number - CRD42024588533 & Keywords)* |
| **Introduction** |  |  |
| [3. Rationale](https:/resources.equator-network.org/reporting-guidelines/prisma/items/rationale.html?utm_source=prisma&utm_medium=checklist&utm_campaign=1_1) | Describe the rationale for the review in the context of existing knowledge. | Introduction - paragraphs 1–7 *(context on ICF framework, prevalence/impact of conditions, need for unified classification, gap in the literature)* |
| [4. Objectives](https:/resources.equator-network.org/reporting-guidelines/prisma/items/objectives.html?utm_source=prisma&utm_medium=checklist&utm_campaign=1_1) | Provide an explicit statement of the objective(s) or question(s) the review addresses. | Introduction - Objective - paragraph 8 *(explicit aim of identifying relevant ICF categories across components)* |
| **Methods** |  |  |
| [5. Eligibility criteria](https:/resources.equator-network.org/reporting-guidelines/prisma/items/eligibility-criteria.html?utm_source=prisma&utm_medium=checklist&utm_campaign=1_1) | Specify the inclusion and exclusion criteria for the review and how studies were grouped for the syntheses. | Methods - *Eligibility Criteria and Data Extraction* - paragraphs 3–4 |
| [6. Information sources](https:/resources.equator-network.org/reporting-guidelines/prisma/items/information-sources.html?utm_source=prisma&utm_medium=checklist&utm_campaign=1_1) | Specify all databases, registers, websites, organisations, reference lists and other sources searched or consulted to identify studies. Specify the date when each source was last searched or consulted. | Methods - *Literature Search* -paragraph 2 |
| [7. Search](https:/resources.equator-network.org/reporting-guidelines/prisma/items/search.html?utm_source=prisma&utm_medium=checklist&utm_campaign=1_1) | Present the full search strategies for all databases, registers and websites, including any filters and limits used. | Methods - *Literature Search* - paragraph 2+ Supplementary Materials |
| [8. Selection Process](https:/resources.equator-network.org/reporting-guidelines/prisma/items/selection-process.html?utm_source=prisma&utm_medium=checklist&utm_campaign=1_1) | Specify the methods used to decide whether a study met the inclusion criteria of the review, including how many reviewers screened each record and each report retrieved, whether they worked independently, and, if applicable, details of automation tools used in the process. | Methods - *Eligibility Criteria and Data Extraction* - paragraph 5 |
| [9. Data collection process](https:/resources.equator-network.org/reporting-guidelines/prisma/items/data-collection-process.html?utm_source=prisma&utm_medium=checklist&utm_campaign=1_1) | Specify the methods used to collect data from reports, including how many reviewers collected data from each report, whether they worked independently, any processes for obtaining or confirming data from study investigators, and if applicable, details of automation tools used in the process. | Methods - *Eligibility Criteria and Data Extraction* - paragraphs 6–7 |
| 10. Data Items |  |  |
| [10a. Outcomes](https:/resources.equator-network.org/reporting-guidelines/prisma/items/data-items-outcomes.html?utm_source=prisma&utm_medium=checklist&utm_campaign=1_1) | List and define all outcomes for which data were sought. Specify whether all results that were compatible with each outcome domain in each study were sought (e.g. for all measures, time points, analyses), and if not, the methods used to decide which results to collect. | Methods - *Eligibility Criteria and Data Extraction* - paragraphs 3 and 7 |
| [10b. Other Variables](https:/resources.equator-network.org/reporting-guidelines/prisma/items/data-items-other-variables.html?utm_source=prisma&utm_medium=checklist&utm_campaign=1_1) | List and define all other variables for which data were sought (e.g. participant and intervention characteristics, funding sources). Describe any assumptions made about any missing or unclear information. | Methods - *Eligibility Criteria and Data Extraction* - paragraph 7 |
| [11. Risk of bias in individual studies](https:/resources.equator-network.org/reporting-guidelines/prisma/items/risk-of-bias-in-individual-studies.html?utm_source=prisma&utm_medium=checklist&utm_campaign=1_1) | Specify the methods used to assess risk of bias in the included studies, including details of the tool(s) used, how many reviewers assessed each study and whether they worked independently, and if applicable, details of automation tools used in the process. | Methods - *Risk of Bias Assessment* - paragraph 8 |
| [12. Effect measures](https:/resources.equator-network.org/reporting-guidelines/prisma/items/effect-measures.html?utm_source=prisma&utm_medium=checklist&utm_campaign=1_1) | Specify for each outcome the effect measure(s) (e.g. risk ratio, mean difference) used in the synthesis or presentation of results. | Methods - *Data Synthesis and Presentation* - paragraph 9 *(Not applicable: no effect measures computed due to descriptive nature of synthesis)* |
| 13. Synthesis Methods |  |  |
| [13a. Deciding which studies were eligible for each synthesis](https:/resources.equator-network.org/reporting-guidelines/prisma/items/synthesis-methods-eligibility.html?utm_source=prisma&utm_medium=checklist&utm_campaign=1_1) | Describe the processes used to decide which studies were eligible for each synthesis (such as tabulating the study intervention characteristics and comparing against the planned groups for each synthesis described in item 5. | Methods - *Data Synthesis and Presentation* - paragraph 9 |
| [13b. Data preparation methods](https:/resources.equator-network.org/reporting-guidelines/prisma/items/synthesis-methods-data-preparation.html?utm_source=prisma&utm_medium=checklist&utm_campaign=1_1) | Describe any methods required to prepare the data for presentation or synthesis, such as handling of missing summary statistics, or data conversions. | Methods - *Data Synthesis and Presentation* - paragraph 9 |
| [13c. Methods for tabulating or displaying results](https:/resources.equator-network.org/reporting-guidelines/prisma/items/synthesis-methods-tabulating-or-displaying-results.html?utm_source=prisma&utm_medium=checklist&utm_campaign=1_1) | Describe any methods used to tabulate or visually display results of individual studies and syntheses. | Methods - *Data Synthesis and Presentation* - paragraph 9 |
| [13d. Synthesis methods](https:/resources.equator-network.org/reporting-guidelines/prisma/items/synthesis-methods-synthesis-methods.html?utm_source=prisma&utm_medium=checklist&utm_campaign=1_1) | Describe any methods used to synthesize results and provide a rationale for the choice(s). If meta-analysis was performed, describe the model(s), method(s) to identify the presence and extent of statistical heterogeneity, and software package(s) used. | Methods - *Data Synthesis and Presentation* - paragraph 9 |
| [13e. Methods for exploring heterogeneity](https:/resources.equator-network.org/reporting-guidelines/prisma/items/synthesis-methods-exploring-heterogeneity.html?utm_source=prisma&utm_medium=checklist&utm_campaign=1_1) | Describe any methods used to explore possible causes of heterogeneity among study results (e.g. subgroup analysis, meta-regression). | Methods - *Data Synthesis and Presentation* - paragraph 9 (Not applicable: no meta-analysis planned) |
| [13f. Sensitivity analyses](https:/resources.equator-network.org/reporting-guidelines/prisma/items/synthesis-methods-sensitivity-analyses.html?utm_source=prisma&utm_medium=checklist&utm_campaign=1_1) | Describe any sensitivity analyses conducted to assess robustness of the synthesized results. | Methods - *Data Synthesis and Presentation* - paragraph 9 *(Not applicable: no quantitative synthesis)* |
| [14. Reporting bias assessment](https:/resources.equator-network.org/reporting-guidelines/prisma/items/reporting-bias-assessment.html?utm_source=prisma&utm_medium=checklist&utm_campaign=1_1) | Describe any methods used to assess risk of bias due to missing results in a synthesis (arising from reporting biases). | Methods - *Data Synthesis and Presentation* - paragraph 9 |
| [15. Certainty assessment](https:/resources.equator-network.org/reporting-guidelines/prisma/items/certainty-assessment.html?utm_source=prisma&utm_medium=checklist&utm_campaign=1_1) | Describe any methods used to assess certainty (or confidence) in the body of evidence for an outcome. | Methods - *Data Synthesis and Presentation* - paragraph 9 (Not applicable: descriptive mapping only) |
| **Results** |  |  |
| 16. Study Selection |  |  |
| [16a. Results of the search and selection process](https:/resources.equator-network.org/reporting-guidelines/prisma/items/study-selection-search-results.html?utm_source=prisma&utm_medium=checklist&utm_campaign=1_1) | Describe the results of the search and selection process, from the number of records identified in the search to the number of studies included in the review, ideally using a flow diagram. | Results - *Study selection* - paragraph 1 + Figure 1 (PRISMA flow diagram) |
| [16b. Excluded studies](https:/resources.equator-network.org/reporting-guidelines/prisma/items/study-selection-excluded-studies.html?utm_source=prisma&utm_medium=checklist&utm_campaign=1_1) | Cite studies that might appear to meet the inclusion criteria, but which were excluded, and explain why they were excluded. | Results - *Study selection* - paragraph 1 *(reasons described; individual citations not required due to large number)* |
| [17. Study characteristics](https:/resources.equator-network.org/reporting-guidelines/prisma/items/study-characteristics.html?utm_source=prisma&utm_medium=checklist&utm_campaign=1_1) | Cite each included study and present its characteristics. | Results - *Study characteristics* - paragraph 2 + Table 1 |
| [18. Risk of bias in studies](https:/resources.equator-network.org/reporting-guidelines/prisma/items/risk-of-bias-in-studies.html?utm_source=prisma&utm_medium=checklist&utm_campaign=1_1) | Present assessments of risk of bias for each included study. | Risk of Bias Assessment  Appendix |
| [19. Results of individual studies](https:/resources.equator-network.org/reporting-guidelines/prisma/items/results-of-individual-studies.html?utm_source=prisma&utm_medium=checklist&utm_campaign=1_1) | For all outcomes, present, for each study: (a) summary statistics for each group (where appropriate) and (b) an effect estimate and its precision (e.g. confidence/credible interval), ideally using structured tables or plots. | (Not applicable: no effect estimates; narrative synthesis only — justified in Methods) |
| 20. Results of Synthesis |  |  |
| [20a. Summary of studies](https:/resources.equator-network.org/reporting-guidelines/prisma/items/results-of-syntheses-summary-of-studies.html?utm_source=prisma&utm_medium=checklist&utm_campaign=1_1) | For each synthesis, briefly summarise the characteristics and risk of bias among contributing studies. | Results - *Methodology used in included studies* - paragraph 3 |
| [20b. Statistical results](https:/resources.equator-network.org/reporting-guidelines/prisma/items/results-of-syntheses-statistical-results.html?utm_source=prisma&utm_medium=checklist&utm_campaign=1_1) | Present results of all statistical syntheses conducted. If meta-analysis was done, present for each the summary estimate and its precision (e.g. confidence/credible interval) and measures of statistical heterogeneity. If comparing groups, describe the direction of the effect. | Not applicable: no meta-analysis conducted |
| [20c. Heterogeneity](https:/resources.equator-network.org/reporting-guidelines/prisma/items/results-of-syntheses-heterogeneity.html?utm_source=prisma&utm_medium=checklist&utm_campaign=1_1) | Present results of all investigations of possible causes of heterogeneity among study results. | Not applicable: no quantitative synthesis |
| [20d. Sensitivity analyses](https:/resources.equator-network.org/reporting-guidelines/prisma/items/results-of-syntheses-sensitivity-analyses.html?utm_source=prisma&utm_medium=checklist&utm_campaign=1_1) | Present results of all sensitivity analyses conducted to assess the robustness of the synthesized results. | Not applicable: no quantitative synthesis |
| [21. Risk of reporting biases in syntheses](https:/resources.equator-network.org/reporting-guidelines/prisma/items/risk-of-reporting-biases-in-syntheses.html?utm_source=prisma&utm_medium=checklist&utm_campaign=1_1) | Present assessments of risk of bias due to missing results (arising from reporting biases) for each synthesis assessed. | Methods - *Data Synthesis and Presentation* (paragraph 9) + implicitly reflected in Results — no change needed |
| [22. Certainty of evidence](https:/resources.equator-network.org/reporting-guidelines/prisma/items/certainty-of-evidence.html?utm_source=prisma&utm_medium=checklist&utm_campaign=1_1) | Present assessments of certainty (or confidence) in the body of evidence for each outcome assessed. | Methods - *Data Synthesis and Presentation* (paragraph 9) *(Not applicable: not a certainty-graded review)* |
| **Discussion** |  |  |
| 23. Discussion |  |  |
| [23a. General interpretation of the results](https:/resources.equator-network.org/reporting-guidelines/prisma/items/discussion-general-interpretation.html?utm_source=prisma&utm_medium=checklist&utm_campaign=1_1) | Provide a general interpretation of the results in the context of other evidence. | Discussion - *Summary of Key Findings* - paragraph 1 + *Comparison with Existing Evidence* - paragraph 2 |
| [23b. Limitations of included evidence](https:/resources.equator-network.org/reporting-guidelines/prisma/items/discussion-limitations-of-included-evidence.html?utm_source=prisma&utm_medium=checklist&utm_campaign=1_1) | Discuss any limitations of the evidence included in the review. | Discussion - *Strengths and Limitations* - paragraph 6 |
| [23c. Limitations of the review processes](https:/resources.equator-network.org/reporting-guidelines/prisma/items/discussion-limitations-of-review-process.html?utm_source=prisma&utm_medium=checklist&utm_campaign=1_1) | Discuss any limitations of the review processes used. | Discussion - *Strengths and Limitations* - paragraph 6 |
| [23d. Implications](https:/resources.equator-network.org/reporting-guidelines/prisma/items/discussion-implications.html?utm_source=prisma&utm_medium=checklist&utm_campaign=1_1) | Discuss implications of the results for practice, policy, and future research. | Discussion - *Implications for Practice and Policy* - paragraph 3–4 + *Future Research Directions* - paragraph 7 |
| **Other Information** |  |  |
| 24. Registration and Protocol |  |  |
| [24a. Registration](https:/resources.equator-network.org/reporting-guidelines/prisma/items/registration-and-protocol-registration.html?utm_source=prisma&utm_medium=checklist&utm_campaign=1_1) | Provide registration information for the review, including register name and registration number, or state that the review was not registered. | Methods  paragraph 1 |
| [24b. Protocol](https:/resources.equator-network.org/reporting-guidelines/prisma/items/registration-and-protocol-protocol.html?utm_source=prisma&utm_medium=checklist&utm_campaign=1_1) | Indicate where the review protocol can be accessed, or state that a protocol was not prepared. | Methods  paragraph 1 (PROSPERO link publicly available) |
| [24c. Amendments](https:/resources.equator-network.org/reporting-guidelines/prisma/items/registration-and-protocol-amendments.html?utm_source=prisma&utm_medium=checklist&utm_campaign=1_1) | Describe and explain any amendments to information provided at registration or in the protocol. | Ethics and Declarations - Not applicable: no amendments were made after registration |
| [25. Support](https:/resources.equator-network.org/reporting-guidelines/prisma/items/support.html?utm_source=prisma&utm_medium=checklist&utm_campaign=1_1) | Describe sources of financial or non-financial support for the review, and the role of the funders or sponsors in the review. | Ethics and Declarations - *Funding* - paragraph 4 |
| [26. Competing Interests](https:/resources.equator-network.org/reporting-guidelines/prisma/items/competing-interests.html?utm_source=prisma&utm_medium=checklist&utm_campaign=1_1) | Declare any competing interests of review authors. | Ethics and Declarations - *Competing interests* - paragraph 6 |
| [27. Availability of data, code, and other materials](https:/resources.equator-network.org/reporting-guidelines/prisma/items/availability-of-materials.html?utm_source=prisma&utm_medium=checklist&utm_campaign=1_1) | Report which of the following are publicly available and where they can be found: template data collection forms; data extracted from included studies; data used for all analyses; analytic code; any other materials used in the review. | Ethics and Declarations - *Data availability* - paragraph 6 + Supplementary Materials (Supplementary Table 1) |

## 1 How to specify where content is

Tell the reader where they can find information. E.g.,

- Results; paragraph 2
- Methods, Participants; paragraphs 1 & 2.
- Table 3
- Supplement B, para. 4

If you have chosen not to describe an item, explain why. You can do this in the checklist, or as a note below it.

You can describe items in the article body, or in tables, figures, or supplementary materials, and should prioritize items you feel are most important to your intended audience. The order of items in your manuscript does not need to match the order of items in this checklist. You can decide how best to structure your work.

## 2 How to cite

Describe how you used PRISMA 2020 at the end of your Methods section, referencing the resources you used e.g.,

‘We used the PRISMA 2020 reporting guideline(1) to draft this manuscript, and the PRISMA 2020 reporting checklist(2) when editing, included in supplement A’

If you use a reporting checklist, remember to include it as a supplement when publishing so that readers can easily find information and see how you have interpreted the guidance.

1. Page MJ, McKenzie JE, Bossuyt PM, Boutron I, Hoffmann TC, Mulrow CD, et al. The PRISMA 2020 statement: An updated guideline for reporting systematic reviews. PLOS Medicine [Internet]. 2021 Mar;18(3):e1003583. Available from: <https://journals.plos.org/plosmedicine/article?id=10.1371/journal.pmed.1003583>

2. Page MJ, McKenzie JE, Bossuyt PM, Boutron I, Hoffmann TC, Mulrow CD, et al. The PRISMA 2020 reporting checklist. In: Harwood J, Albury C, Beyer J de, Schlüssel M, Collins G, editors. The EQUATOR network reporting guideline platform [Internet]. The UK EQUATOR Centre; 2025. Available from: [https:/resources.equator-network.org/reporting-guidelines/prisma/prisma-checklist.docx](https://https:/resources.equator-network.org/reporting-guidelines/prisma/prisma-checklist.docx)
