# Supplementary material for: Identification of International Classification of Functioning, Disability and Health (ICF) codes most frequently used to describe functioning in children: a systematic review
Source: BMJ Paediatr Open. 2026 Jun 19;10(1):e004292. doi: 10.1136/bmjpo-2025-004292 (PMC13289332; doi:10.1136/bmjpo-2025-004292)
Supplement: online supplemental file 2 [file bmjpo-10-1-s002.docx]

## Appendices

### Search strategies

PUBMED

("International Classification of Functioning, Disability and Health"[Mesh] OR "Disability Evaluation"[Mesh] OR "ICF") AND ("core set" OR "core sets" OR "code set" OR "code sets")

("International Classification of Functioning, Disability and Health"[Mesh] OR "Disability Evaluation"[Mesh] OR "ICF") AND ("core set" OR "core sets" OR "code set" OR "code sets") AND ("Child"[Mesh] OR "Adolescent"[Mesh] OR "Child, Preschool" OR "Infant" OR "Infant, Newborn"[Mesh])

SCOPUS

("International Classification of Functioning, Disability and Health" OR "Disability Evaluation" OR "ICF") AND ("core set" OR "core sets" OR "code set" OR "code sets") AND ("Child*" OR "Adolescent*" OR "Preschool" OR "Infant*" OR "Newborn*")

WoS

("International Classification of Functioning, Disability and Health" OR "Disability Evaluation" OR "ICF") AND ("core set" OR "core sets" OR "code set" OR "code sets") AND ("Child*" OR "Adolescent*" OR "Preschool" OR "Infant*" OR "Newborn*")

CINAHL

("International Classification of Functioning, Disability and Health" OR "Disability Evaluation" OR "ICF") AND ("core set" OR "core sets" OR "code set" OR "code sets") AND ("Child*" OR "Adolescent*" OR "Preschool" OR "Infant*" OR "Newborn*")

### **Risk of Bias Assessment using the Mixed Methods Appraisal Tool (MMAT)**

MMAT version 2018 was used to assess methodological quality across included studies. Criteria were applied according to study type (qualitative, quantitative, mixed methods). 'Yes' indicates criterion met; 'No' indicates not met; 'Cannot tell' indicates unclear reporting. All studies were considered low risk of bias.

|  | **First author** | Luo | Güeita-Rodríguez | Bölte | Bölte | Ellingsen | Bölte | Bölte | Schiariti |
| --- | --- | --- | --- | --- | --- | --- | --- | --- | --- |
|  | **Year** | 2022 | 2019 | 2024 | 2019 | 2024 | 2024 | 2018 | 2015 |
| **SCREENING**  **QUESTIONS** | S1. Are there clear research questions? | Yes | Yes | Yes | Yes | Yes | Yes | Yes | Yes |
|  | S2. Do the collected data allow to address the research questions? | Yes | Yes | Yes | Yes | Yes | Yes | Yes | Yes |
| 1. **QUALITATIVE**   **STUDIES** | 1.1. Is the qualitative approach appropriate to answer the research question? | Yes | Yes | Yes | Yes | Yes | Yes | Yes | Yes |
|  | 1.2. Are the qualitative data collection methods adequate to address the research question? | Yes | Yes | Yes | Yes | Yes | Yes | Yes | Yes |
|  | 1.3. Are the findings adequately derived from the data? | Yes | Yes | Yes | Yes | Yes | Yes | Yes | Yes |
|  | 1.4. Is the interpretation of results sufficiently substantiated by data? | Yes | Yes | Yes | Yes | Yes | Yes | Yes | Yes |
|  | 1.5. Is there coherence between qualitative data sources, collection, analysis and interpretation? | Yes | Yes | Yes | Yes | Yes | Yes | Yes | Yes |
| **4. QUANTITATIVE DESCRIPTIVE STUDIES** | 4.1. Is the sampling strategy relevant to address the research question? | Yes | Yes |  | Yes |  |  | Yes | Yes |
|  | 4.2. Is the sample representative of the target population? | Yes | Yes |  | Yes |  |  | Yes | Yes |
|  | 4.3. Are the measurements appropriate? | Yes | Yes |  | Yes |  |  | Yes | Yes |
|  | 4.4. Is the risk of nonresponse bias low? | Yes | Yes |  | Yes |  |  | Yes | Yes |
|  | 4.5. Is the statistical analysis appropriate to answer the research question? | Yes | Yes |  | Yes |  |  | Yes | Yes |
| **5. MIXED METHODS STUDIES** | 5.1. Is there an adequate rationale for using a mixed methods design to address the research question? | Yes | Yes |  | Yes |  |  | Yes | Yes |
|  | 5.2. Are the different components of the study effectively integrated to answer the research question? | Yes | Yes |  | Yes |  |  | Yes | Yes |
|  | 5.3. Are the outputs of the integration of qualitative and quantitative components adequately interpreted? | Yes | Yes |  | Yes |  |  | Yes | Yes |
|  | 5.4. Are divergences and inconsistencies between quantitative and qualitative results adequately addressed? | Yes | Yes |  | Yes |  |  | Yes | Yes |
|  | 5.5. Do the different components of the study adhere to the quality criteria of each tradition of the methods involved? | Yes | Yes |  | Yes |  |  | Yes | Yes |
| **COMMENTS** | | **Low risk of bias** | **Low risk of bias** | **Low risk of bias** | **Low risk of bias** | **Low risk of bias** | **Low risk of bias** | **Low risk of bias** | **Low risk of bias** |
