# Supplementary material for: Identification of International Classification of Functioning, Disability and Health (ICF) codes most frequently used to describe functioning in children: a systematic review
Source: BMJ Paediatr Open. 2026 Jun 19;10(1):e004292. doi: 10.1136/bmjpo-2025-004292 (PMC13289332; doi:10.1136/bmjpo-2025-004292)
Supplement: online supplemental file 3 [file bmjpo-10-1-s003.docx]

| **Core set** | | Congenital Heart  desease | Aquatic Physical  Therapy | | | | | Autism | | | Autism | | | | ADHA | | | | ADHA | | | Cerebral Palsy | | | | | | | | | Developmental | | | | | |  |
| --- | --- | --- | --- | --- | --- | --- | --- | --- | --- | --- | --- | --- | --- | --- | --- | --- | --- | --- | --- | --- | --- | --- | --- | --- | --- | --- | --- | --- | --- | --- | --- | --- | --- | --- | --- | --- | --- |
|  |  | 3 – 6 years | Comprehensive 0 - 18y | Common Brief | 0 - <6 years | >6 – <14 years | >14 – <18 years | Comprehensive/ lifespan | Comprehensive 0-5y | Comprehensive 6 – 16y | Comprehensive/ lifespan | Brief/ lifespan | Brief/ 0 – 5 years | Brief 6 16 years | Comprehensive/ lifespan | Brief/ lifespan | Brief/ 0 – 5 years | Brief 6ª-16 years | Comprehensive/ lifespan | 0 – 5 years | 6 - 16 years | Brief 0 -18 years | Brief 0 – 6 years | | Brief 6 - 14 years | | Brief 14 - 18 years | | Comprehensive 0 - 18y | | 0 – 2 years | | 3 – 5 years | | 6 – 12 years | 13 – 17 years |  |
| **BODY FUNCTIONS** | |  |  |  |  |  |  |  |  |  |  |  |  |  |  |  |  |  |  |  |  |  |  | |  | |  | |  | |  | |  | |  |  | |
| b110 - consciousness functions | |  | ■ |  |  |  |  |  |  |  |  |  |  |  |  |  |  |  |  |  |  |  |  | |  | |  | |  | | ■ | |  | |  |  | 2 |
| b114 - Orientation functions | |  | ■ |  |  | ■ | ■ | ■ | ■ | ■ | ■ | ■ | ■ | ■ |  |  |  |  |  |  |  |  |  | |  | |  | |  | |  | |  | |  |  | 10 |
| b117 - Intellectual functions | |  |  |  |  |  |  | ■ |  | ■ | ■ | ■ | ■ | ■ |  |  |  |  |  |  |  | ■ | ■ | | ■ | | ■ | | ■ | |  | |  | |  |  | 11 |
| b122 - Global psychosocial functions | | ■ |  |  |  |  |  | ■ | ■ | ■ | ■ | ■ | ■ | ■ | ■ |  |  |  |  |  |  |  |  | |  | |  | |  | |  | |  | |  |  | 9 |
| b125 - Dispositions and intra-personal functions | |  |  |  |  |  |  | ■ | ■ | ■ | ■ | ■ | ■ | ■ |  | ■ | ■ | ■ | ■ | ■ | ■ |  |  | |  | |  | |  | |  | |  | |  |  | 13 |
| b126 - Temperament and personality functions | |  |  |  |  |  |  | ■ | ■ | ■ | ■ | ■ | ■ | ■ |  |  |  |  | ■ | ■ | ■ |  |  | |  | |  | | ■ | |  | |  | | ■ | ■ | 13 |
| b130 - Energy and drive functions | |  |  |  |  |  |  | ■ | ■ | ■ | ■ | ■ | ■ | ■ | ■ | ■ | ■ | ■ | ■ | ■ | ■ |  |  | |  | |  | |  | |  | |  | | ■ | ■ | 16 |
| b1301 - Motivation | |  | ■ |  | ■ |  | ■ |  |  |  |  |  |  |  |  |  |  |  |  |  |  |  |  | | ■ | | ■ | | ■ | |  | |  | |  |  | 6 |
| b1302 - Appetite | | ■ |  |  |  |  |  |  |  |  |  |  |  |  |  |  |  |  |  |  |  |  |  | |  | |  | |  | |  | |  | |  |  | 1 |
| b134 - Sleep functions | | ■ |  |  |  |  |  | ■ | ■ | ■ | ■ | ■ | ■ | ■ | ■ | ■ | ■ | ■ | ■ | ■ | ■ | ■ | ■ | | ■ | | ■ | | ■ | |  | | ■ | | ■ | ■ | 23 |
| b140 - Attention functions | |  | ■ |  |  |  |  | ■ | ■ | ■ | ■ | ■ | ■ | ■ | ■ | ■ | ■ | ■ | ■ | ■ | ■ |  |  | | ■ | |  | | ■ | |  | | ■ | | ■ | ■ | 20 |
| b144 - Memory functions | |  |  |  |  |  |  | ■ | ■ | ■ | ■ | ■ | ■ | ■ |  |  |  |  | ■ | ■ | ■ |  |  | |  | |  | |  | |  | | ■ | | ■ | ■ | 13 |
| b147 - Psychomotor functions | | ■ | ■ |  | ■ |  |  | ■ | ■ | ■ | ■ | ■ | ■ | ■ | ■ | ■ | ■ | ■ | ■ | ■ | ■ |  |  | |  | |  | |  | |  | |  | | ■ |  | 17 |
| b152 - Emotional functions (G) | | ■ |  |  |  |  |  | ■ | ■ | ■ | ■ | ■ | ■ | ■ | ■ | ■ | ■ | ■ | ■ | ■ | ■ |  |  | |  | |  | | ■ | | ■ | |  | | ■ | ■ | 19 |
| b156 - Perceptual functions | |  | ■ |  |  |  |  | ■ | ■ | ■ | ■ | ■ | ■ | ■ |  |  |  |  |  |  |  |  |  | |  | |  | | ■ | |  | | ■ | |  |  | 10 |
| b160 - Thought functions | |  |  |  |  |  |  | ■ | ■ | ■ | ■ | ■ | ■ | ■ |  |  |  |  |  |  |  |  |  | |  | |  | |  | |  | |  | | ■ | ■ | 9 |
| b163 - Basic cognitive functions | |  | ■ |  |  |  |  |  |  |  |  |  |  |  |  |  |  |  |  |  |  |  |  | |  | |  | | ■ | |  | |  | | ■ |  | 3 |
| b164 - Higher-level cognitive functions | |  |  |  |  |  |  | ■ |  | ■ | ■ | ■ | ■ | ■ | ■ | ■ | ■ | ■ | ■ |  | ■ |  |  | |  | | ■ | | ■ | |  | |  | | ■ | ■ | 16 |
| b167 - Mental functions of language | |  |  |  |  |  |  | ■ | ■ | ■ | ■ | ■ | ■ | ■ |  |  |  |  |  |  |  | ■ | ■ | | ■ | | ■ | | ■ | |  | |  | | ■ | ■ | 14 |
| b210 - Seeing functions | |  | ■ |  | ■ |  |  | ■ | ■ | ■ |  |  |  |  |  |  |  |  | ■ | ■ | ■ | ■ | ■ | | ■ | | ■ | | ■ | | ■ | |  | |  |  | 14 |
| b2152 - Functions of external muscles of the eye | |  |  |  |  |  |  |  |  |  |  |  |  |  |  |  |  |  |  |  |  |  |  | |  | |  | | ■ | |  | |  | |  |  | 1 |
| b230 - Hearing functions | |  |  |  |  |  |  | ■ | ■ | ■ |  |  |  |  |  |  |  |  | ■ | ■ | ■ |  | ■ | |  | |  | | ■ | | ■ | |  | |  |  | 9 |
| b235 - Vestibular functions | |  | ■ | ■ | ■ | ■ | ■ |  |  |  |  |  |  |  |  |  |  |  |  |  |  |  |  | |  | |  | |  | |  | |  | |  |  | 5 |
| b250 - Taste functions | |  |  |  |  |  |  | ■ | ■ | ■ |  |  |  |  |  |  |  |  | ■ | ■ | ■ |  |  | |  | |  | |  | |  | |  | |  |  | 6 |
| b255 - Smell Functions | |  |  |  |  |  |  | ■ | ■ | ■ |  |  |  |  |  |  |  |  | ■ | ■ | ■ |  |  | |  | |  | |  | |  | |  | |  |  | 6 |
| b265 - Touch function | |  | ■ |  |  |  |  | ■ | ■ | ■ | ■ |  |  |  |  |  |  |  | ■ | ■ | ■ |  |  | |  | |  | |  | | ■ | |  | |  |  | 8 |
| b270 - Sensory functions related to temperature and other stimuli | |  |  |  |  |  |  | ■ | ■ | ■ | ■ |  | ■ |  |  |  |  |  | ■ | ■ | ■ |  |  | |  | |  | |  | |  | |  | |  |  | 8 |
| b280 - Sensation of pain | |  | ■ | ■ | ■ | ■ | ■ | ■ | ■ | ■ |  |  |  |  |  |  |  |  | ■ | ■ | ■ | ■ | ■ | | ■ | | ■ | | ■ | | ■ | |  | | ■ |  | 18 |
| b298 - Sensory functions and pain, other specified | |  |  |  |  |  |  | ■ | ■ | ■ |  |  |  |  |  |  |  |  | ■ | ■ | ■ |  |  | |  | |  | |  | |  | |  | |  |  | 6 |
| b310- Voice end producing sounds and speech | |  |  |  |  |  |  |  |  |  |  |  |  |  |  |  |  |  |  |  |  |  |  | |  | |  | |  | | ■ | | ■ | |  |  | 2 |
| b320 - Articulation functions | |  |  |  |  |  |  |  |  |  |  |  |  |  |  |  |  |  |  |  |  |  |  | |  | |  | | ■ | |  | |  | | ■ |  | 2 |
| b330 - Fluency and rhythm of speech functions | |  |  |  |  |  |  | ■ | ■ | ■ | ■ | ■ | ■ | ■ |  |  |  |  |  |  |  |  |  | |  | |  | |  | |  | |  | | ■ |  | 8 |
| b410 - Heart funcyions | | ■ | ■ |  |  |  |  |  |  |  |  |  |  |  |  |  |  |  |  |  |  |  |  | |  | |  | |  | | ■ | | ■ | | ■ | ■ | 6 |
| b430 - Hematological system functions | | ■ |  |  |  |  |  |  |  |  |  |  |  |  |  |  |  |  |  |  |  |  |  | |  | |  | |  | |  | |  | |  |  | 1 |
| b435 - Immunological system functions | | ■ |  |  |  |  |  |  |  |  |  |  |  |  |  |  |  |  |  |  |  |  |  | |  | |  | |  | | ■ | | ■ | | ■ | ■ | 5 |
| b440 - Respiration functions | | ■ | ■ | ■ | ■ | ■ | ■ |  |  |  |  |  |  |  |  |  |  |  |  |  |  |  |  | |  | |  | | ■ | | ■ | | ■ | | ■ | ■ | 11 |
| b445 - Respiratory muscle functions | |  | ■ | ■ | ■ | ■ | ■ |  |  |  |  |  |  |  |  |  |  |  |  |  |  |  |  | |  | |  | | ■ | |  | |  | |  |  | 6 |
| b4501 - Transportation of airways mucus | |  |  |  |  |  |  |  |  |  |  |  |  |  |  |  |  |  |  |  |  |  |  | |  | |  | | ■ | |  | |  | |  |  | 1 |
| b455 - Exercise tolerance functions | | ■ | ■ |  |  | ■ | ■ |  |  |  |  |  |  |  |  |  |  |  |  |  |  |  |  | |  | |  | | ■ | |  | |  | |  |  | 5 |
| b460- Sensationswith cardiovascular ande respiratory functions | |  |  |  |  |  |  |  |  |  |  |  |  |  |  |  |  |  |  |  |  |  |  | |  | |  | |  | |  | |  | |  | ■ | 1 |
| b510 - Ingestion functions | | ■ |  |  |  |  |  |  |  |  |  |  |  |  |  |  |  |  |  |  |  |  |  | |  | |  | | ■ | | ■ | | ■ | |  |  | 4 |
| b515 - Deigestive functions | | ■ |  |  |  |  |  |  |  |  |  |  |  |  |  |  |  |  |  |  |  |  |  | |  | |  | |  | | ■ | |  | |  |  | 2 |
| b525 - Defecation functions | |  |  |  |  |  |  |  |  |  |  |  |  |  |  |  |  |  |  |  |  |  |  | |  | |  | | ■ | |  | | ■ | |  |  | 2 |
| b530 - Weight maintenance functions | | ■ |  |  |  |  |  |  |  |  |  |  |  |  |  |  |  |  |  |  |  |  |  | |  | |  | | ■ | |  | |  | | ■ | ■ | 4 |
| b545 - Water, mineral and eleetrolyte balance functions | | ■ |  |  |  |  |  |  |  |  |  |  |  |  |  |  |  |  |  |  |  |  |  | |  | |  | |  | |  | |  | |  |  | 1 |
| b560 - Growth maintenance functions | | ■ |  |  |  |  |  |  |  |  |  |  |  |  |  |  |  |  |  |  |  |  |  | |  | |  | |  | | ■ | | ■ | | ■ | ■ | 5 |
| b620 - Urination functions | |  |  |  |  |  |  |  |  |  |  |  |  |  |  |  |  |  |  |  |  |  |  | |  | |  | | ■ | |  | | ■ | |  |  | 2 |
| b650 - Menstrual functions | |  |  |  |  |  |  |  |  |  |  |  |  |  |  |  |  |  |  |  |  |  |  | |  | |  | |  | |  | |  | |  | ■ | 1 |
| b710 - Mobility of joint functions | |  | ■ | ■ | ■ | ■ | ■ |  |  |  |  |  |  |  |  |  |  |  |  |  |  | ■ | ■ | | ■ | | ■ | | ■ | |  | |  | |  |  | 10 |
| b715 - Stability of joint functions | |  | ■ |  |  | ■ | ■ |  |  |  |  |  |  |  |  |  |  |  |  |  |  |  |  | |  | |  | | ■ | |  | |  | |  |  | 4 |
| b730 - Muscle power functions | |  | ■ |  |  | ■ | ■ |  |  |  |  |  |  |  |  |  |  |  |  |  |  |  |  | |  | |  | | ■ | |  | |  | |  |  | 4 |
| b735 - Muscle tone functions | |  | ■ | ■ | ■ | ■ | ■ |  |  |  |  |  |  |  |  |  |  |  |  |  |  | ■ | ■ | | ■ | | ■ | | ■ | | ■ | | ■ | | ■ |  | 13 |
| b740 - Muscle endurance functions | |  | ■ |  |  | ■ | ■ |  |  |  |  |  |  |  |  |  |  |  |  |  |  |  |  | |  | |  | | ■ | |  | |  | |  |  | 4 |
| b750 - Motor reflex functions | |  | ■ |  |  |  |  |  |  |  |  |  |  |  |  |  |  |  |  |  |  |  |  | |  | |  | |  | | ■ | |  | |  |  | 2 |
| b755 - Involuntary movement reaction functions | |  | ■ |  |  |  |  |  |  |  |  |  |  |  |  |  |  |  |  |  |  |  |  | |  | |  | | ■ | | ■ | | ■ | |  |  | 4 |
| b760 - Control of voluntary movement functions | |  | ■ |  | ■ | ■ | ■ | ■ | ■ | ■ | ■ | ■ | ■ | ■ | ■ |  | ■ | ■ | ■ | ■ | ■ | ■ | ■ | | ■ | | ■ | | ■ | |  | | ■ | | ■ |  | 24 |
| b761- Spontaneouse moveents | |  |  |  |  |  |  |  |  |  |  |  |  |  |  |  |  |  |  |  |  |  |  | |  | |  | |  | | ■ | |  | |  |  | 1 |
| b765 - Involuntary movement functions | |  |  |  |  |  |  | ■ | ■ | ■ | ■ | ■ | ■ | ■ |  |  |  |  |  |  |  |  |  | |  | |  | | ■ | |  | |  | |  |  | 8 |
| b770 - Gait pattern functions | |  | ■ |  |  |  |  |  |  |  |  |  |  |  |  |  |  |  |  |  |  |  |  | |  | |  | | ■ | |  | |  | |  |  | 2 |
| b780 - Sensations related to muscles and movement functions | |  | ■ |  |  |  |  |  |  |  |  |  |  |  |  |  |  |  |  |  |  |  |  | |  | |  | |  | |  | |  | |  |  | 1 |
| b810 - Protective functions of the skin | |  |  |  |  |  |  |  |  |  |  |  |  |  |  |  |  |  |  |  |  |  |  | |  | |  | | ■ | |  | |  | |  |  | 1 |
| **ACTIVITIES AND PARTICIPATION** | | | | | | | | | | | | | | | | | | | | | | | | | | | | | | | | | | | | | |
| d110 - Watching | |  | ■ |  |  |  |  | ■ | ■ |  | ■ |  | ■ | ■ |  |  |  |  |  |  |  |  |  | |  | |  | | ■ | | ■ | |  | |  |  | 8 |
| d115 - Listening | |  | ■ |  |  |  |  | ■ | ■ | ■ | ■ |  | ■ | ■ |  |  |  |  |  |  |  |  |  | |  | |  | | ■ | |  | |  | |  |  | 8 |
| d120 - Other pursposeful sensing | |  |  |  |  |  |  |  |  |  |  |  |  |  |  |  |  |  |  |  |  |  |  | |  | |  | | ■ | | ■ | |  | |  |  | 2 |
| d130 - Copying | |  | ■ |  |  |  |  | ■ | ■ | ■ | ■ |  | ■ | ■ |  |  |  |  |  |  |  |  |  | |  | |  | | ■ | |  | |  | |  |  | 8 |
| d131 - Learning through actions with objects | |  | ■ |  |  |  |  |  |  |  |  |  |  |  |  |  |  |  |  |  |  |  |  | |  | |  | | ■ | | ■ | | ■ | |  |  | 4 |
| d132 - Acquiring information | |  |  |  |  |  |  | ■ | ■ | ■ |  | ■ | ■ | ■ |  |  |  |  |  |  |  |  |  | |  | |  | |  | |  | |  | |  |  | 6 |
| d133 - Acquiring language | |  |  |  |  |  |  |  |  |  |  |  |  |  |  |  |  |  |  |  |  |  | ■ | |  | |  | | ■ | | ■ | | ■ | |  |  | 4 |
| d137 - Acquiring concepts | |  |  |  |  |  |  | ■ | ■ | ■ | ■ |  | ■ | ■ |  |  |  |  |  |  |  |  |  | |  | |  | | ■ | |  | | ■ | |  |  | 8 |
| d140 - Learning to read | |  |  |  |  |  |  | ■ |  | ■ | ■ |  |  | ■ |  |  |  |  | ■ |  | ■ |  |  | |  | |  | | ■ | |  | |  | | ■ |  | 8 |
| d145 - Learning to write | |  |  |  |  |  |  | ■ |  | ■ | ■ |  |  | ■ |  |  |  |  | ■ |  | ■ |  |  | |  | |  | | ■ | |  | |  | | ■ |  | 8 |
| d150-Learning to calculate | |  |  |  |  |  |  |  |  |  |  |  |  |  |  |  |  |  |  |  |  |  |  | |  | |  | |  | |  | |  | | ■ |  | 1 |
| d155 - Acquiring skills | |  | ■ |  |  |  |  | ■ | ■ | ■ | ■ | ■ | ■ | ■ |  |  |  |  |  |  |  |  | ■ | |  | |  | | ■ | |  | |  | |  |  | 10 |
| d160 - Focusing attention | | ■ | ■ |  |  |  |  | ■ | ■ | ■ | ■ | ■ | ■ | ■ | ■ | ■ | ■ | ■ | ■ | ■ | ■ |  |  | |  | |  | | ■ | |  | |  | | ■ |  | 18 |
| d161 - Directing attention | |  | ■ |  |  |  |  | ■ | ■ | ■ | ■ |  | ■ | ■ | ■ | ■ | ■ | ■ | ■ | ■ | ■ |  |  | |  | |  | |  | |  | |  | | ■ |  | 15 |
| d163 - Thinking | |  |  |  |  |  |  | ■ |  | ■ | ■ |  |  | ■ |  |  |  |  | ■ |  | ■ |  |  | |  | |  | |  | |  | |  | | ■ | ■ | 8 |
| d166 - Reading | |  |  |  |  |  |  | ■ |  | ■ | ■ |  |  |  | ■ |  |  | ■ | ■ |  | ■ |  |  | |  | |  | | ■ | |  | |  | |  |  | 8 |
| d170 - Writing | |  |  |  |  |  |  |  |  |  | ■ |  |  |  | ■ |  |  | ■ | ■ |  | ■ |  |  | |  | |  | | ■ | |  | |  | | ■ | ■ | 8 |
| d172 - Calculating | |  |  |  |  |  |  |  |  |  |  |  |  |  | ■ |  |  | ■ | ■ |  | ■ |  |  | |  | |  | | ■ | |  | |  | | ■ | ■ | 7 |
| d175 - Solving problems | |  | ■ |  |  |  |  | ■ |  | ■ | ■ |  |  | ■ | ■ | ■ | ■ | ■ |  |  | ■ |  |  | | ■ | | ■ | | ■ | |  | |  | | ■ | ■ | 15 |
| d177 - Making decisions | |  |  |  |  |  |  | ■ |  | ■ | ■ |  |  | ■ | ■ | ■ | ■ | ■ |  |  | ■ |  |  | |  | |  | | ■ | |  | |  | | ■ | ■ | 12 |
| d210 - Undertaking a single task | |  | ■ |  |  |  |  | ■ | ■ | ■ | ■ | ■ | ■ | ■ | ■ |  | ■ | ■ |  | ■ | ■ |  |  | |  | |  | |  | |  | | ■ | |  |  | 14 |
| d220 - Undertaking multiple tasks | |  | ■ |  |  |  |  | ■ |  | ■ | ■ | ■ | ■ | ■ | ■ | ■ | ■ | ■ |  |  | ■ |  |  | |  | |  | | ■ | |  | |  | | ■ |  | 14 |
| d230 - Carrying out daily routine (G) | |  |  |  |  |  |  | ■ | ■ | ■ | ■ | ■ | ■ | ■ | ■ | ■ | ■ | ■ |  | ■ | ■ |  |  | | ■ | |  | | ■ | |  | |  | | ■ |  | 16 |
| d240 - Handling stress and other psychological demands | |  |  |  |  |  |  | ■ | ■ | ■ | ■ | ■ | ■ | ■ | ■ | ■ | ■ | ■ |  | ■ | ■ |  |  | |  | |  | |  | |  | |  | | ■ | ■ | 15 |
| d250 - Managing one's own behaviour | |  |  |  |  |  |  | ■ | ■ | ■ | ■ | ■ | ■ | ■ | ■ | ■ | ■ | ■ |  | ■ | ■ |  |  | |  | | ■ | | ■ | |  | |  | |  |  | 15 |
| d310 - Communicating with - receiving - spoken messages | |  |  |  |  |  |  | ■ | ■ | ■ | ■ | ■ | ■ | ■ |  |  |  |  |  | ■ | ■ |  |  | |  | |  | | ■ | | ■ | |  | | ■ |  | 12 |
| d315 - Communicating with - receiving - nonverbal messages | |  | ■ |  |  |  |  | ■ | ■ | ■ | ■ | ■ | ■ | ■ |  |  |  |  |  |  |  |  |  | |  | |  | |  | |  | |  | | ■ | ■ | 10 |
| d330 - Speaking | |  | ■ |  |  |  |  | ■ | ■ | ■ | ■ | ■ | ■ | ■ |  |  |  |  |  |  |  |  |  | |  | |  | | ■ | |  | |  | | ■ |  | 10 |
| d331 - Pre-talking | |  |  |  |  |  |  | ■ | ■ |  | ■ |  | ■ |  |  |  |  |  |  |  |  |  |  | |  | |  | | ■ | | ■ | |  | |  |  | 6 |
| d335 - Producing nonverbal messages | |  | ■ |  |  |  |  | ■ | ■ |  | ■ |  | ■ |  |  |  |  |  |  |  |  |  |  | |  | |  | | ■ | |  | |  | |  |  | 6 |
| d350 - Conversation | |  |  |  |  |  |  | ■ | ■ | ■ | ■ |  |  | ■ | ■ |  | ■ | ■ |  | ■ | ■ |  |  | | ■ | |  | | ■ | |  | | ■ | | ■ | ■ | 15 |
| d360 - Using communication devices and techniques | |  |  |  |  |  |  | ■ |  | ■ | ■ |  | ■ |  |  |  |  |  |  |  |  |  |  | |  | |  | | ■ | |  | |  | |  | ■ | 6 |
| d410 - Changing basic body position | |  | ■ | ■ | ■ | ■ | ■ |  |  |  |  |  |  |  |  |  |  |  |  |  |  |  |  | |  | |  | | ■ | | ■ | |  | |  |  | 7 |
| d415 - Maintaining a body position | |  | ■ | ■ | ■ | ■ | ■ |  |  |  |  |  |  |  |  |  |  |  |  |  |  | ■ | ■ | | ■ | | ■ | | ■ | | ■ | |  | |  |  | 11 |
| d420 - Transferring oneself | |  | ■ | ■ | ■ | ■ | ■ |  |  |  |  |  |  |  |  |  |  |  |  |  |  |  |  | |  | |  | | ■ | |  | |  | |  |  | 6 |
| d430 - Lifting and carrying objects | |  | ■ |  |  |  |  |  |  |  |  |  |  |  |  |  |  |  |  |  |  |  |  | |  | |  | | ■ | |  | |  | |  |  | 2 |
| d435 - Moving objects with lower extremities | |  | ■ |  |  |  |  |  |  |  |  |  |  |  |  |  |  |  |  |  |  |  |  | |  | |  | |  | |  | |  | |  |  | 1 |
| d440 - Fine hand use | |  |  |  |  |  |  | ■ | ■ |  |  |  |  |  | ■ |  | ■ |  |  | ■ |  | ■ | ■ | | ■ | | ■ | | ■ | |  | |  | |  |  | 10 |
| d445 - Hand and arm use | |  | ■ | ■ | ■ | ■ | ■ |  |  |  |  |  |  |  |  |  |  |  |  |  |  |  |  | |  | |  | | ■ | | ■ | | ■ | |  |  | 8 |
| d450 - Walking | |  | ■ | ■ | ■ | ■ | ■ |  |  |  |  |  |  |  |  |  |  |  |  |  |  | ■ | ■ | | ■ | | ■ | | ■ | |  | | ■ | |  |  | 11 |
| d455 - Moving around | |  | ■ | ■ | ■ | ■ | ■ |  |  |  |  |  |  |  |  |  |  |  |  |  |  |  |  | |  | |  | | ■ | | ■ | | ■ | |  |  | 8 |
| d460 - Moving around in different locations | |  |  |  |  |  |  |  |  |  |  |  |  |  |  |  |  |  |  |  |  | ■ | ■ | | ■ | | ■ | | ■ | |  | |  | |  |  | 5 |
| d465 - Moving around using equipment | |  | ■ |  |  |  |  |  |  |  |  |  |  |  |  |  |  |  |  |  |  |  |  | |  | |  | | ■ | |  | | ■ | |  |  | 3 |
| d470 - Using transportation | |  |  |  |  |  |  | ■ |  | ■ | ■ |  |  | ■ |  |  |  |  |  |  |  |  |  | |  | |  | | ■ | |  | |  | |  | ■ | 6 |
| d475 - Driving | |  |  |  |  |  |  | ■ |  | ■ | ■ |  |  |  | ■ |  |  |  |  |  | ■ |  |  | |  | |  | |  | |  | |  | |  |  | 5 |
| d510 - Washing oneself | |  | ■ |  |  |  |  | ■ | ■ | ■ | ■ |  |  | ■ |  |  |  |  |  |  |  |  |  | |  | |  | | ■ | |  | |  | | ■ |  | 8 |
| d520 - Caring for body parts | |  |  |  |  |  |  | ■ |  | ■ | ■ |  |  |  | ■ |  |  | ■ |  |  | ■ |  |  | |  | |  | | ■ | |  | |  | | ■ | ■ | 9 |
| d530 - Toileting | |  |  |  |  |  |  | ■ | ■ | ■ | ■ |  | ■ | ■ |  |  |  |  |  |  |  | ■ | ■ | | ■ | | ■ | | ■ | |  | | ■ | |  |  | 12 |
| d540 - Dressing | |  | ■ |  |  |  |  | ■ | ■ | ■ | ■ |  |  | ■ |  |  |  |  |  |  |  |  |  | |  | |  | | ■ | |  | |  | | ■ |  | 8 |
| d550 - Eating | |  |  |  |  |  |  | ■ | ■ | ■ | ■ |  | ■ |  |  |  |  |  |  | ■ | ■ | ■ | ■ | | ■ | | ■ | | ■ | |  | |  | |  |  | 12 |
| d560 - Drinking | |  |  |  |  |  |  |  |  |  |  |  |  |  |  |  |  |  |  |  |  |  |  | |  | |  | | ■ | |  | |  | |  |  | 1 |
| d570 - Looking after one's health | |  |  |  |  |  |  | ■ | ■ | ■ | ■ | ■ | ■ | ■ | ■ |  |  | ■ |  | ■ | ■ |  |  | |  | | ■ | | ■ | |  | |  | |  | ■ | 14 |
| d571 - Looking after one's safety | |  |  |  |  |  |  | ■ |  | ■ | ■ | ■ | ■ | ■ | ■ | ■ | ■ | ■ |  | ■ | ■ |  |  | |  | |  | |  | |  | |  | | ■ | ■ | 14 |
| d620 - Acquisition of goods and services | |  |  |  |  |  |  | ■ |  |  | ■ |  |  |  | ■ |  |  |  |  |  |  |  |  | |  | |  | |  | |  | |  | |  |  | 3 |
| d630 - Preparing meals | |  |  |  |  |  |  | ■ |  |  | ■ |  |  |  | ■ |  |  |  |  |  |  |  |  | |  | |  | | ■ | |  | |  | |  | ■ | 5 |
| d640 - Doing housework | |  |  |  |  |  |  | ■ |  |  | ■ |  |  |  | ■ |  |  |  |  |  |  |  |  | |  | |  | | ■ | |  | |  | |  | ■ | 5 |
| d650 - Caring for household objects | |  |  |  |  |  |  | ■ |  |  | ■ |  |  |  |  |  |  |  | ■ |  |  |  |  | |  | |  | |  | |  | |  | |  |  | 3 |
| d660 - Assisting others | |  |  |  |  |  |  | ■ |  | ■ | ■ |  |  |  |  |  |  |  | ■ |  | ■ |  |  | |  | |  | |  | |  | |  | |  | ■ | 6 |
| d710 - Basic interpersonal interactions | |  | ■ |  |  |  |  | ■ | ■ | ■ | ■ | ■ | ■ | ■ | ■ | ■ | ■ | ■ | ■ | ■ | ■ | ■ | ■ | | ■ | | ■ | | ■ | |  | | ■ | | ■ | ■ | 23 |
| d720 - Complex interpersonal interactions | |  |  |  |  |  |  | ■ | ■ | ■ | ■ | ■ | ■ | ■ | ■ | ■ | ■ | ■ | ■ | ■ | ■ |  |  | |  | | ■ | | ■ | |  | |  | | ■ | ■ | 18 |
| d730 - Relating with strangers | |  |  |  |  |  |  | ■ |  | ■ | ■ |  |  | ■ |  |  |  |  |  |  |  |  |  | |  | |  | |  | |  | |  | |  |  | 4 |
| d740 - Formal relationships | |  |  |  |  |  |  | ■ |  | ■ | ■ |  |  |  | ■ |  |  | ■ | ■ |  | ■ |  |  | |  | |  | |  | |  | |  | |  |  | 7 |
| d750 - Informal social relationships | |  |  |  |  |  |  | ■ |  | ■ | ■ |  |  | ■ | ■ |  | ■ | ■ | ■ |  | ■ |  |  | |  | |  | | ■ | |  | |  | |  |  | 10 |
| d760 - Family relationships | |  |  |  |  |  |  | ■ |  | ■ | ■ | ■ | ■ | ■ | ■ | ■ | ■ | ■ | ■ |  | ■ | ■ | ■ | | ■ | | ■ | | ■ | |  | |  | | ■ | ■ | 19 |
| d770 - Intimate relationships | |  |  |  |  |  |  | ■ |  |  | ■ |  |  |  | ■ |  |  |  | ■ |  |  |  |  | |  | |  | | ■ | |  | |  | |  | ■ | 6 |
| d815 - Preschool education | |  |  |  |  |  |  | ■ | ■ |  |  |  |  |  |  |  |  |  | ■ | ■ |  |  |  | |  | |  | | ■ | |  | | ■ | |  |  | 6 |
| d820 - School education | | ■ |  |  |  |  |  | ■ |  | ■ | ■ | ■ | ■ | ■ | ■ | ■ | ■ | ■ | ■ | ■ | ■ |  |  | | ■ | | ■ | | ■ | |  | |  | | ■ |  | 18 |
| d825 - Vocational training | |  |  |  |  |  |  | ■ |  |  | ■ |  |  |  | ■ |  |  |  | ■ |  |  |  |  | |  | |  | |  | |  | |  | |  |  | 4 |
| d830 - Higher education | |  |  |  |  |  |  | ■ |  |  | ■ |  |  |  | ■ |  |  |  | ■ |  |  |  |  | |  | |  | |  | |  | |  | |  |  | 4 |
| d845 - Acquiring, keeping and terminating a job | |  |  |  |  |  |  | ■ |  |  | ■ |  |  |  | ■ |  |  |  | ■ |  |  |  |  | |  | | ■ | | ■ | |  | |  | |  |  | 6 |
| d850 - Remunerative employment (G) | |  |  |  |  |  |  | ■ |  |  | ■ |  |  |  | ■ |  |  |  | ■ |  |  |  |  | |  | |  | |  | |  | |  | |  |  | 4 |
| d860 - Basic economic transactions | |  |  |  |  |  |  | ■ |  |  | ■ |  |  |  |  |  |  |  | ■ |  |  |  |  | |  | |  | | ■ | |  | |  | |  | ■ | 5 |
| d870 - Economic self-sufficiency | |  |  |  |  |  |  | ■ |  |  | ■ |  |  |  | ■ |  |  |  | ■ |  |  |  |  | |  | |  | |  | |  | |  | |  |  | 4 |
| d880 - Engagement in play | | ■ | ■ |  |  |  |  | ■ | ■ | ■ | ■ | ■ | ■ | ■ | ■ |  | ■ | ■ | ■ | ■ | ■ |  | ■ | |  | |  | | ■ | | ■ | | ■ | | ■ |  | 20 |
| d910 - Community life | |  |  |  |  |  |  | ■ |  |  | ■ |  |  |  |  |  |  |  | ■ |  |  |  |  | |  | |  | | ■ | |  | |  | |  |  | 4 |
| d920 - Recreation and leisure | |  |  |  |  |  |  | ■ | ■ | ■ | ■ | ■ | ■ | ■ | ■ | ■ | ■ | ■ | ■ | ■ | ■ |  |  | | ■ | | ■ | | ■ | |  | |  | | ■ | ■ | 19 |
| d9200 - Play | |  | ■ |  | ■ | ■ |  |  |  |  |  |  |  |  |  |  |  |  |  |  |  |  |  | |  | |  | |  | |  | |  | |  |  | 3 |
| d930 - Religion and Spirituality | |  |  |  |  |  |  | ■ |  |  |  |  |  |  |  |  |  |  | ■ |  | ■ |  |  | |  | |  | |  | |  | |  | |  |  | 3 |
| d940 - Human rights | |  |  |  |  |  |  | ■ |  |  | ■ |  |  |  |  |  |  |  | ■ |  |  |  |  | |  | |  | |  | |  | |  | |  |  | 3 |
| **ENVIRONMENTAL FACTORS** | |  |  |  |  |  |  |  |  |  |  |  |  |  |  |  |  |  |  |  |  |  |  | |  | |  | |  | |  | |  | |  |  | |
| e110 - Products or substances for personal consumption | |  |  |  |  |  |  | ■ | ■ | ■ | ■ | ■ | ■ | ■ | ■ | ■ | ■ | ■ | ■ | ■ | ■ |  |  | |  | |  | | ■ | | ■ | | ■ | | ■ | ■ | 19 |
| e115 - Products and technology for personal use in daily living | |  |  |  |  |  |  | ■ | ■ | ■ | ■ | ■ | ■ | ■ | ■ | ■ | ■ | ■ | ■ | ■ | ■ | ■ | ■ | | ■ | | ■ | | ■ | | ■ | | ■ | | ■ | ■ | 23 |
| e120 - Products and technology for personal indoor and outdoor mobility and transportation | |  |  |  |  |  |  |  |  |  |  |  |  |  |  |  |  |  |  |  |  | ■ | ■ | | ■ | | ■ | | ■ | |  | | ■ | |  | ■ | 7 |
| e125 - Products and technology for communication | |  |  |  |  |  |  | ■ | ■ | ■ | ■ | ■ | ■ | ■ | ■ |  |  | ■ | ■ |  | ■ | ■ | ■ | | ■ | | ■ | | ■ | |  | | ■ | | ■ | ■ | 19 |
| e130 - Products and technology for education | |  |  |  |  |  |  | ■ |  | ■ | ■ | ■ | ■ | ■ | ■ |  |  | ■ | ■ |  | ■ |  |  | | ■ | |  | | ■ | |  | | ■ | | ■ | ■ | 15 |
| e140 - Products and technology for culture, recreation and sport | |  |  |  |  |  |  |  |  |  |  |  |  |  |  |  |  |  |  |  |  |  |  | | ■ | |  | | ■ | |  | | ■ | |  | ■ | 4 |
| e150 - Design, construction and building products and technology of buildings for public use | |  |  |  |  |  |  |  |  |  |  |  |  |  |  |  |  |  |  |  |  | ■ | ■ | | ■ | | ■ | | ■ | |  | | ■ | | ■ | ■ | 8 |
| e155 - Design, construction and building products and technology of buildings for private use | |  |  |  |  |  |  |  |  |  |  |  |  |  |  |  |  |  |  |  |  |  |  | |  | |  | | ■ | |  | |  | |  |  | 1 |
| e160 - Products and technology of land development | |  |  |  |  |  |  |  |  |  |  |  |  |  |  |  |  |  |  |  |  |  |  | |  | |  | | ■ | |  | |  | |  |  | 1 |
| e165 - Assets | |  |  |  |  |  |  |  |  |  |  |  |  |  |  |  |  |  |  |  |  |  |  | |  | |  | | ■ | |  | |  | |  |  | 1 |
| e240 - Light | |  |  |  |  |  |  | ■ | ■ | ■ | ■ |  | ■ |  | ■ |  |  | ■ | ■ | ■ | ■ |  |  | |  | |  | |  | |  | |  | |  |  | 10 |
| e250 - Sound | |  |  |  |  |  |  | ■ | ■ | ■ | ■ |  | ■ | ■ | ■ |  | ■ | ■ | ■ | ■ | ■ |  |  | |  | |  | |  | |  | |  | |  |  | 12 |
| e310 - Immediate family | |  | ■ |  |  |  |  | ■ | ■ | ■ | ■ | ■ | ■ | ■ | ■ | ■ | ■ | ■ | ■ | ■ | ■ | ■ | ■ | | ■ | | ■ | | ■ | | ■ | | ■ | | ■ | ■ | 24 |
| e315 - Extended family | |  |  |  |  |  |  | ■ | ■ | ■ | ■ | ■ | ■ | ■ | ■ | ■ | ■ | ■ | ■ | ■ | ■ |  |  | |  | |  | | ■ | |  | | ■ | |  |  | 16 |
| e320 - Friends | |  |  |  |  |  |  | ■ | ■ | ■ | ■ |  |  | ■ | ■ | ■ | ■ | ■ | ■ | ■ | ■ | ■ | ■ | | ■ | | ■ | | ■ | |  | | ■ | | ■ | ■ | 20 |
| e325 - Acquaintances, peers, colleagues, neighbours and community members | |  |  |  |  |  |  | ■ | ■ | ■ | ■ | ■ | ■ | ■ | ■ |  |  | ■ | ■ | ■ | ■ |  |  | |  | |  | | ■ | |  | |  | |  | ■ | 14 |
| e330 - People in positions of authority | |  |  |  |  |  |  | ■ | ■ | ■ | ■ | ■ | ■ | ■ | ■ | ■ | ■ | ■ | ■ | ■ | ■ |  |  | |  | |  | | ■ | |  | |  | |  | ■ | 16 |
| e340 - Personal care providers and personal assistants | |  |  |  |  |  |  | ■ | ■ | ■ | ■ | ■ | ■ |  | ■ |  | ■ |  | ■ | ■ | ■ |  |  | |  | |  | | ■ | | ■ | | ■ | |  | ■ | 15 |
| e350 - Domestic animals | |  |  |  |  |  |  | ■ |  |  |  |  |  |  |  |  |  |  | ■ |  | ■ |  |  | |  | |  | |  | |  | |  | |  |  | 4 |
| e355 - Health professionals | |  | ■ |  |  |  |  | ■ | ■ | ■ | ■ | ■ | ■ | ■ | ■ | ■ | ■ | ■ | ■ | ■ | ■ |  | ■ | |  | |  | | ■ | | ■ | | ■ | |  |  | 19 |
| e360 - Other professionals | |  |  |  |  |  |  | ■ | ■ | ■ | ■ | ■ | ■ | ■ | ■ |  |  |  |  |  |  |  |  | |  | |  | |  | |  | |  | |  |  | 8 |
| e410 - Individual attitudes of immediate family members | | ■ |  |  |  |  |  | ■ | ■ | ■ | ■ | ■ | ■ | ■ | ■ | ■ | ■ | ■ | ■ | ■ | ■ |  | ■ | |  | |  | | ■ | |  | | ■ | | ■ | ■ | 20 |
| e415 - Individual attitudes of extended family members | |  |  |  |  |  |  | ■ | ■ | ■ | ■ | ■ | ■ | ■ | ■ | ■ | ■ | ■ | ■ | ■ | ■ |  |  | |  | |  | | ■ | |  | | ■ | |  |  | 16 |
| e420 - Individual attitudes of friends | |  |  |  |  |  |  | ■ | ■ | ■ | ■ |  |  |  | ■ |  |  | ■ | ■ | ■ | ■ |  |  | |  | | ■ | | ■ | |  | |  | | ■ | ■ | 13 |
| e425 - Individual attitudes of acquaintances, peers, colleagues, neighbours and community members | |  |  |  |  |  |  | ■ | ■ | ■ |  |  |  |  | ■ |  |  |  | ■ | ■ | ■ |  |  | |  | |  | | ■ | |  | |  | |  |  | 8 |
| e430 - Individual attitudes of people in positions of authority | |  |  |  |  |  |  | ■ | ■ | ■ | ■ | ■ | ■ | ■ | ■ | ■ | ■ | ■ | ■ | ■ | ■ |  |  | |  | |  | | ■ | |  | |  | |  |  | 15 |
| e440 - Individual attitudes of personal care providers and personal assistants | |  |  |  |  |  |  | ■ | ■ | ■ |  |  |  |  | ■ |  | ■ |  | ■ | ■ | ■ |  |  | |  | |  | | ■ | |  | |  | |  |  | 9 |
| e450 - Individual attitudes of health professionals | | ■ |  |  |  |  |  | ■ | ■ | ■ | ■ | ■ | ■ | ■ | ■ | ■ | ■ | ■ | ■ | ■ | ■ |  |  | |  | |  | | ■ | |  | |  | |  |  | 16 |
| e455 - Individual attitudes of other professionals | |  |  |  |  |  |  | ■ | ■ | ■ | ■ |  |  | ■ | ■ |  |  |  | ■ | ■ | ■ |  |  | |  | |  | |  | |  | |  | |  |  | 9 |
| e460 - Societal attitudes | |  |  |  |  |  |  | ■ | ■ | ■ | ■ | ■ | ■ | ■ | ■ | ■ | ■ |  | ■ | ■ | ■ | ■ | ■ | | ■ | | ■ | | ■ | |  | |  | |  |  | 18 |
| e465 - Social norms, practices and ideologies | |  |  |  |  |  |  | ■ | ■ | ■ | ■ | ■ | ■ | ■ | ■ | ■ | ■ | ■ | ■ | ■ | ■ |  |  | |  | |  | | ■ | |  | |  | |  |  | 15 |
| e525 - Housing services, systems and policies | |  |  |  |  |  |  | ■ |  |  | ■ |  |  |  |  |  |  |  | ■ |  |  |  |  | |  | |  | | ■ | |  | |  | |  |  | 4 |
| e535 - Communication services, systems and policies | |  |  |  |  |  |  | ■ |  | ■ | ■ |  |  | ■ |  |  |  |  | ■ |  | ■ |  |  | |  | |  | |  | |  | |  | |  |  | 6 |
| e540 - Transportation services, systems and policies | |  |  |  |  |  |  |  |  |  |  |  |  |  |  |  |  |  |  |  |  |  |  | |  | | ■ | | ■ | |  | |  | |  | ■ | 3 |
| e550 - Legal services, systems and policies | |  |  |  |  |  |  | ■ | ■ | ■ | ■ | ■ | ■ | ■ |  |  |  |  | ■ | ■ | ■ |  |  | |  | |  | | ■ | |  | |  | |  |  | 11 |
| e555 - Associations and organizational services, systems and policies | |  |  |  |  |  |  |  |  |  |  |  |  |  |  |  |  |  |  |  |  |  |  | |  | |  | | ■ | |  | |  | |  |  | 1 |
| e560 - Media services, systems and policies | |  |  |  |  |  |  | ■ |  | ■ | ■ |  |  |  |  |  |  |  | ■ |  | ■ |  |  | |  | |  | | ■ | |  | |  | |  |  | 6 |
| e570 - Social security services, systems and policies | |  |  |  |  |  |  | ■ | ■ | ■ | ■ | ■ | ■ | ■ | ■ | ■ | ■ | ■ | ■ | ■ | ■ |  |  | |  | |  | | ■ | | ■ | | ■ | | ■ | ■ | 19 |
| e575 - General social support services, systems and policies | |  |  |  |  |  |  | ■ | ■ | ■ | ■ | ■ | ■ | ■ | ■ | ■ | ■ | ■ | ■ | ■ |  |  |  | |  | |  | | ■ | |  | |  | |  |  | 14 |
| e580 - Health services, systems and policies | | ■ | ■ |  |  |  |  | ■ | ■ | ■ | ■ | ■ | ■ | ■ | ■ | ■ | ■ | ■ | ■ | ■ | ■ | ■ | ■ | | ■ | | ■ | | ■ | | ■ | | ■ | | ■ | ■ | 25 |
| e585 - Education and training services, systems and policies | |  |  |  |  |  |  | ■ | ■ | ■ | ■ | ■ | ■ | ■ | ■ | ■ | ■ | ■ | ■ | ■ | ■ |  |  | | ■ | | ■ | | ■ | |  | |  | |  |  | 17 |
| e590 - Labour and employment services, systems and policies | |  |  |  |  |  |  | ■ |  |  | ■ | ■ | ■ | ■ | ■ |  |  |  | ■ |  |  |  |  | |  | |  | | ■ | |  | |  | |  |  | 8 |
| **BODY STRUCTURES** |  |  |  |  |  |  |  |  |  |  |  |  |  |  |  |  |  |  |  |  |  |  |  |  | |  | |  | |  | |  | |  |  |  | |
| s110 - Structure of brain | |  |  |  |  |  |  |  |  |  | ■ |  |  |  |  |  |  |  |  |  |  | ■ | ■ | | ■ | | ■ | | ■ | |  | |  | |  |  | 6 |
| s320 - Structure of mouth | |  |  |  |  |  |  |  |  |  |  |  |  |  |  |  |  |  |  |  |  |  |  | |  | |  | | ■ | |  | |  | |  |  | 1 |
| S 710 - Structure of head and neck region | |  | ■ |  |  |  |  |  |  |  |  |  |  |  |  |  |  |  |  |  |  |  |  | |  | |  | |  | |  | |  | |  |  | 1 |
| s720 - Structure of shoulder region | |  | ■ |  |  |  |  |  |  |  |  |  |  |  |  |  |  |  |  |  |  |  |  | |  | |  | |  | |  | |  | |  |  | 1 |
| s730 - Structure of upper extremity | |  | ■ |  |  |  |  |  |  |  |  |  |  |  |  |  |  |  |  |  |  |  |  | |  | |  | | ■ | |  | |  | |  |  | 2 |
| s740 - Structure of pelvic region | |  | ■ |  |  |  |  |  |  |  |  |  |  |  |  |  |  |  |  |  |  |  |  | |  | |  | |  | |  | |  | |  |  | 1 |
| s750 - Structure of lower extremity | |  | ■ |  |  |  |  |  |  |  |  |  |  |  |  |  |  |  |  |  |  |  |  | |  | |  | | ■ | |  | |  | |  |  | 2 |
| s760 - Structure of trunk | |  | ■ | ■ | ■ | ■ | ■ |  |  |  |  |  |  |  |  |  |  |  |  |  |  |  |  | |  | |  | | ■ | |  | |  | |  |  | 6 |
| s7600m - Structure of Vertebral Column | |  | ■ |  |  |  |  |  |  |  |  |  |  |  |  |  |  |  |  |  |  |  |  | |  | |  | |  | |  | |  | |  |  | 1 |
| s770 - Additional musculoskeletal structures related to movement | |  | ■ |  |  |  |  |  |  |  |  |  |  |  |  |  |  |  |  |  |  |  |  | |  | |  | |  | |  | |  | |  |  | 1 |
| s7700 - Bones | |  |  |  |  |  |  |  |  |  |  |  |  |  |  |  |  |  |  |  |  |  |  | |  | |  | | ■ | |  | |  | |  |  | 1 |
| s7703 - Extra-articular ligaments, fasciae, extramuscular aponeuroses, retinacula, septa, bursae, | |  |  |  |  |  |  |  |  |  |  |  |  |  |  |  |  |  |  |  |  |  |  | |  | |  | | ■ | |  | |  | |  |  | 1 |

Table 3: ICF Codes Across Included Core Sets (N=1665)
