# Supplementary material for: Identification of International Classification of Functioning, Disability and Health (ICF) codes most frequently used to describe functioning in children: a systematic review
Source: BMJ Paediatr Open. 2026 Jun 19;10(1):e004292. doi: 10.1136/bmjpo-2025-004292 (PMC13289332; doi:10.1136/bmjpo-2025-004292)
Supplement: Abstract translation 1 [file bmjpo-10-1-s004.pdf]

Objetivos:

Identificar e sintetizar as categorias da Classificação Internacional de Funcionalidade, Incapacidade e Saúde (CIF) mais utilizadas para descrever a funcionalidade infantil em diferentes condições de saúde, informando avaliações e enquadramentos de políticas inclusivos e sensíveis ao contexto.

Desenho:

Revisão sistemática.

Fontes de dados:

Foram pesquisadas as bases de dados PubMed, Scopus, Web of Science e CINAHL desde o seu início até setembro de 2024, de acordo com as orientações Cochrane.

Critérios de elegibilidade:

Foram incluídos estudos com revisão por pares que utilizaram categorias da CIF para avaliar a funcionalidade em crianças (0–18 anos), independentemente da condição de saúde. Os desenhos elegíveis incluíram ensaios clínicos randomizados, estudos observacionais e qualitativos que reportassem Core Sets (CS) da CIF.

Extração e síntese de dados:

Dois revisores realizaram de forma independente a triagem, extração de dados e avaliação do risco de enviesamento utilizando o Mixed Methods Appraisal Tool (MMAT, 2018). Os códigos da CIF foram categorizados por domínio e sintetizados narrativamente.

Resultados:

Oito estudos cumpriram os critérios de inclusão, abrangendo 29 Core Sets da CIF (1665 ocorrências; 194 categorias da CIF após remoção de duplicados). A maioria das ocorrências relacionou-se com Atividades e Participação (40,7%) e Fatores Ambientais (30,6%), seguidos de Funções do Corpo (27,3%) e Estruturas do Corpo (1,4%). Os capítulos principais incluíram aprendizagem e aplicação de conhecimentos, interações interpessoais, apoio e relacionamentos e serviços e políticas.

Conclusões:

Os resultados evidenciam a transição para um modelo biopsicossocial da funcionalidade infantil, enfatizando a participação e contexto ambiental. Sustentam a utilização do enquadramento da CIF na avaliação multidisciplinar e na definição de políticas, bem como informam o desenvolvimento de uma Tabela Nacional de Funcionalidade para Crianças em Portugal.

PROSPERO:

CRD42024588533

Sumário do artigo:

• **O que já se sabe sobre este tema:** A CIF é amplamente utilizada na investigação pediátrica, mas a síntese das categorias mais frequentemente aplicadas em diferentes condições de saúde permanece limitada.

• **O que este estudo acrescenta:** Esta revisão identifica as categorias da CIF mais frequentemente utilizadas nos Core Sets pediátricos, com predominância de Atividades e Participação e Fatores Ambientais.

• **Como este estudo pode influenciar a investigação, prática ou políticas:** Os resultados informam modelos de avaliação inclusivos e independentes do diagnóstico e apoiam abordagens integradas dos cuidados pediátricos, educação e políticas públicas.
