# Supplementary material for: Identification of International Classification of Functioning, Disability and Health (ICF) codes most frequently used to describe functioning in children: a systematic review
Source: BMJ Paediatr Open. 2026 Jun 19;10(1):e004292. doi: 10.1136/bmjpo-2025-004292 (PMC13289332; doi:10.1136/bmjpo-2025-004292)
Supplement: Supplementary file 5 [file bmjpo-2025-004292.reviewer_comments.pdf]

PEER REVIEW HISTORY

BMJ Paediatrics Open publishes all reviews undertaken for accepted manuscripts. Reviewers are asked to complete a checklist review form and are provided with free text boxes to elaborate on their assessment. These free text comments are reproduced below.

ARTICLE DETAILS

**TITLE (PROVISIONAL)**	Identification of International Classification of Functioning, Disability and Health (ICF) Codes Most Frequently Used to Describe Functioning in Children: A Systematic Review
**AUTHORS**	SAntos Vieira Lisboa Bordalo, Idalina Maria Maria Santos Vieira Lisboa<br>Pereira, Carla Martins<br>Fonseca, César<br>Bico, Isabel

VERSION 1 – REVIEW

**REVIEWER NAME**	Ten Napel, Huib
**REVIEWER AFFILIATION**	National Institute for Public Health and the Environment, WHO-FIC CC
**REVIEWER CONFLICT OF INTEREST**	No Conflict of Interest
**AI DISCLOSURE**	No, I have not used AI
**DATE REVIEW RETURNED**	08-Jan-2026

**GENERAL COMMENTS**	This has been a very extensive exercise to collect what is used in Core sets to describe aspects of children's functioning. My compliments for that.<br>I think it not clearly stated why exactly a 'generic' set of codes is required, this is the impression I get from the exercise, and for which purpose and why it could be distilled from existing Core sets, which are by definition 'disease- or disorder' oriented.<br>I would need more context for the purposes it could be used and a stronger argumenation what the benefits would be in specific contexts.<br>It would be useful to explain how and why classes are specific for children in the range from 0-18 and not for human in general. Developmental aspects? markers? So what are the criteria for selecting any item from the Core sets. Is the idea that a set of items would emerge just from these sets? Are there any criteria used to select an ICF class/term?<br>In principle there have been 194 ICF classes identified, not 1665 different classes as stated in the document.<br>I would also like to point out that not all the term are used correctly. There is a confusing use terms like disabilities, disability etc, where probably disorders is meant. I have marked this in the document.<br>This paper has a lot of potential as a first step for development of a common set of codes, specifically relevant to children in a certain context (use), but that would need more refinement of argumentation.

**REVIEWER NAME**	Martins, Elisabete

**REVIEWER AFFILIATION**	Escola Superior de Saúde do Alcoitão, Neurofunctional Physiotherapy
**REVIEWER CONFLICT OF INTEREST**	No Conflict of Interest
**AI DISCLOSURE**	No, I have not used AI
**DATE REVIEW RETURNED**	19-Jan-2026

**GENERAL COMMENTS**	The manuscript rigorously adheres to all methodological steps required for a systematic review, including the formulation of a clear research question, development of a protocol, comprehensive literature search, study selection according to predefined criteria, data extraction and synthesis, and assessment of methodological quality and risk of bias. This systematic review was conducted and reported in accordance with the PRISMA 2020 (Preferred Reporting Items for Systematic Reviews and Meta-Analyses) guidelines to ensure transparency, completeness, and methodological rigour.<br><br>Methodological quality and risk of bias of the included studies were assessed using the Mixed Methods Appraisal Tool (MMAT, version 2018), following its standard guidance. The approach employed to evaluate risk of bias is highly appropriate for mixed-methods studies, fully meeting the methodological requirements of a review of this nature.<br><br>The literature review is well-structured and draws upon recent references, primarily from the last 10 years, ensuring the evidence base is current. The discussion of findings thoughtfully includes comparison with existing evidence and highlights pertinent directions for future research. Furthermore, the appendices provide detailed documentation of all methodological steps, allowing replication of the study, ensuring transparency of the steps undertaken in the development of the study, and facilitating continuity in subsequent research.<br><br>Overall, the manuscript is written in a clear, concise, and objective manner, demonstrates scientific rigor, and is written using language appropriate for the field of science. As a reviewer, I find the study to be methodologically sound and comprehensive, and I have no suggestions for changes or additions. This study represents a valuable contribution to clinical practice and to health policies targeting the pediatric population.

VERSION 1 – AUTHOR RESPONSE

Summary of revisions implemented
Reviewer 1
1. Rationale for a cross-condition (“generic”) paediatric ICF set and intended purpose/benefits:
• We strengthened the introduction to explicitly justify why a cross-condition (“generic”) set is needed alongside condition-specific Core Sets.
• We clarified the intended use cases and benefits in specific contexts where diagnosis may be evolving, uncertain, or multiple and where decisions are guided by functional needs (e.g., early intervention, inclusive education, rehabilitation, community programmes, and service planning).
• We clarified that our systematic review provides an empirical foundation for subsequent consensus/validation work (rather than claiming to deliver a final generic set).
2. Child-specific framing (0–18 years) and developmental rationale:
• We added a developmental explanation describing why functioning domains and contextual

influences are interpreted differently across childhood and adolescence (e.g., age-dependent roles in learning, play, schooling, autonomy, peer relationships; and the decisive role of family/school/services).
• We clarified that ICF categories are not exclusive to children per se, but that their prioritisation and recurrence in paediatric Core Sets is developmentally anchored and therefore informative for paediatric assessment.
3. Criteria/approach for selecting and synthesising ICF categories from Core Sets:
• We expanded the Methods to specify how ICF categories were extracted from each Core Set, how categories were handled (grouping by component/chapter and level where applicable), and how frequency across Core Sets was used to identify the most commonly represented categories.
• We included explicit definitions to avoid ambiguity and to clarify how the "most frequently used" categories were derived from the included Core Sets.
4. Correction of the "1665 vs 194" issue (code instances vs unique categories):
• We corrected and harmonised all reporting to distinguish between:
(i) "code instances across Core Sets" (n=1665; total occurrences summed across Core Sets), and
(ii) (ii) "unique ICF categories after deduplication" (n=194).
• This clarification was implemented consistently across the Abstract, Results, Discussion, and Table 2 (including an explanatory note).
5. Terminology refinement ("disability" vs "disorder/condition"):
• We performed a terminology review throughout the manuscript to ensure alignment with the ICF framework.
• We now use "health condition/disorder/diagnosis" when referring to clinical conditions and reserve "disability/functioning/activity limitation/participation restriction" for functioning-related concepts, avoiding misleading or inconsistent usage.
Reviewer 2
Reviewer 2 reported no suggestions for changes or additions. We appreciate the positive assessment of methodological rigour and reporting.

Files submitted
As requested, we have uploaded:
• A clean revised manuscript ("Main Document"), and
• A marked version highlighting all changes ("Main Document – marked copy"),
along with a detailed point-by-point response to the reviewers.

VERSION 2 – REVIEW

**REVIEWER NAME**	Ten Napel, Huib
**REVIEWER AFFILIATION**	National Institute for Public Health and the Environment, WHO-FIC CC
**REVIEWER CONFLICT OF INTEREST**	No Conflict of Interest
**AI DISCLOSURE**	No, I have not used AI
**DATE REVIEW RETURNED**	24-Mar-2026

**GENERAL COMMENTS**	Please find the review comments in the attached file.

VERSION 2 – AUTHOR RESPONSE

RESPONSE TO REVIEWER 1
1. Data sharing statement
Comment: Data sharing statement.
Response: We thank the reviewer for this suggestion. The data sharing statement has been revised

for clarity and alignment with journal requirements (page 19, lines 1-2). The revised text reads: “All data relevant to this study are included in the article or provided as supplementary information. No additional data are available.”

2. Contributorship statement
Comment: Contributorship statement.
Response: We thank the reviewer for this comment. The contributorship statement has been revised for clarity and consistency with journal style (page 19, lines 10-15).

3. Competing interests statement
Comment: Competing interests statement.
Response: We thank the reviewer for this suggestion. The competing interests statement has been revised to align with journal wording (page 19, line 17). It now reads: “The authors declare no competing interests.”

We have also carefully reviewed the manuscript to ensure consistency throughout, including correct use of ICF codes (see marked copy for details).
We hope the revised manuscript meets the journal’s requirements and we look forward to your consideration.
